# Identification of neuropeptide networks involved in the ecdysis program of a crustacean model: *Carcinus maenas* reveal* s*imilarities and differences to insects that reflect evolutionary divergence in structure and function

**DOI:** 10.1186/s12915-026-02603-w

**Published:** 2026-04-22

**Authors:** Jodi L. Hoppes, David C. Wilcockson, Michael G. B. Hayle, Elsa Larrad, Simon G. Webster

**Affiliations:** 1 School of Natural Sciences, Brambell Laboratories, Bangor University, Bangor, LL57 2UW UK; 2 Department of Life Sciences, Edward Llywd Building, Aberystwyth University, Aberystwyth, SY23 3DA UK

**Keywords:** Ecdysis triggering hormone (ETH), Ecdysis triggering hormone receptor (ETHR), Eclosion hormone (EH), Neuroanatomy, Peptide and transcript expression, Neuropeptide release patterns during ecdysis, Crustaceans, Insects, *Carcinus maenas*

## Abstract

**Abstract:**

**Background:**

Arthropods require periodic molting (ecdysis) for growth. While the neuroendocrine orchestration of ecdysis is well characterized in insects, it remains comparatively poorly understood in crustaceans. In insects, ecdysis-triggering hormone (ETH) from epitracheal cells and eclosion hormone (EH) from the brain initiate and coordinate ecdysis. ETH triggers pre-ecdysis behaviors, while EH amplifies ETH release and promotes neuropeptide secretion of crustacean cardioactive peptide (CCAP), myoinhibitory peptide (MIP) for exuviation. Definitive evidence for functional ETH and EH in crustaceans is lacking. Here, we investigate ETH and EH in the crab *Carcinus maenas* by functionally characterizing the ETH receptor, comprehensively mapping transcript and peptide localization of ETH and EH, and quantifying ETH neurohormone and transcript throughout the molt cycle.

**Results:**

We identified a single CamETH GPCR with high specificity for arthropod ETHs, signaling via calcium and cAMP. ETHR mRNA is expressed in multiple tissues, but ETH is restricted to the central nervous system (CNS) with a complex neuroarchitecture in pericardial organs, adjacent to the branchiocardiac veins. EH is limited to CNS, notably in the eyestalk ganglia. Of note, complex EH immunopositive fibers in the abdominal ganglion, adjacent to CCAP/allatostatin-C (AST-C)/bursicon cells, represent a novel putative neurohemal site. Measurements of ETH mRNA and peptide through the molt cycle showed that transcription peaks in late premolt, while peptide is released during active ecdysis, later than observed in insects.

**Conclusions:**

While crustacean and insect ecdysis share neuroendocrine components, reflecting common ancestry, there is clear divergence in terms of functionality. We propose a new crustacean ecdysis cascade: ETH released from pericardial organs may stimulate EH to initiate CCAP/AST-C and bursicon release from adjacent neurons.

**Supplementary Information:**

The online version contains supplementary material available at 10.1186/s12915-026-02603-w.

## Background

Arthropod growth and development are inextricably linked with periodic shedding of the exoskeleton- molting or ecdysis. This process involves a precisely timed sequence of events, which for insects is composed of three phases: pre-ecdysis, ecdysis, and post ecdysis [1]. The stereotyped behavioral sequences of these have been well studied in various model insects, for example, in lepidopterans: *Manduca sexta* and *Bombyx mori* [2–5], dipterans: *Drosophila melanogaster* [6–8], and Coleoptera: *Tribolium* *castaneum* [9]. An array of hormones is involved in the control of the ecdysis sequence, namely, pre-ecdysis triggering hormone, and ecdysis-triggering hormone (PETH, ETH), eclosion hormone (EH), crustacean cardioactive peptide (CCAP), and bursicon. However, several others also have significant roles, such as kinins, myoinhibitory peptides (MIPs), FMRFamides, and corazonin (CRZ), and involvement in the ecdysis sequence has been comprehensively reviewed [10]. A detailed model showing peptide neuroanatomy and release patterns during the ecdysis program in *Bombyx mori* has recently been reported [11]. In brief, the central command peptides PETH and ETH are released from Inka cells in the paired epitracheal glands near the spiracles of the thoracic and abdominal surfaces [12, 13]. In *Manduca sexta*, these peptides first trigger pre-ecdysis behavior by direct action of the CNS, eliciting fictive motor patterns in isolated CNS preparations that closely mirror in vivo behaviors [14]. While transcription and synthesis of ETH is promoted by high levels of ecdysteroids [15], release of ETH can only occur following the nadir of ecdysteroids seen prior to ecdysis [2]. Additionally, release of CRZ into the hemolymph from neurosecretory terminals in the corpora allata stimulates the release of PETH and ETH from Inka cells, in *M. sexta* and *B. mori *in vitro and in vivo and the cognate receptor is highly expressed in these cells [16].

The insect eclosion hormone (EH) is the second command peptide involved in insect ecdysis [17–19]. This peptide is produced (in many species) by two pairs of ventro-median (*V*_m_) neurons in the brain, which project posteriorly along the ventral nerve cord, to release sites on the proctodeal nerves [20]. Circulating EH acts on Inka cells via a receptor guanylyl cyclase [21] to induce release of ETH, which in turn establishes a feedforward loop which results in the complete depletion of ETH in Inka cells and transitions to the second phase of ecdysis [4, 22]. EH release also causes an increase in cGMP in crustacean cardioactive peptide (CCAP) containing neurons [23, 24] and likely peptide release which initiates the ecdysis motor program [7, 25–27]. Ecdysis initiation is immediately regulated by crustacean cardioactive peptide (CCAP) and myoinhibitory peptides (MIPs) [28]. For *M. sexta*, both these peptides are produced in the interneuron 704 cells (which also express an ETH receptor, ETHR-A) [23, 28, 29]. It has been suggested that MIPs suppress the activity of neurons involved in pre-ecdysis behavior, while CCAP controls the activity of the “ecdysis network” [4]. The final part of the ecdysis program—post ecdysis, involves expansion, plasticization, sclerotization, and hardening of the cuticle which has long been known to be controlled by bursicon [30, 31].

Insects evolved from a crustacean-like ancestor in the early Ordovician 479 mya [32]. The fossil record showing ecdysis in trilobites in the Cambrian era [33], together with the interpretation of an abundant record of ecdysis behaviors in fossil decapod crustaceans [34], suggests that these seem to be identical in extant crabs and crayfish [35], and molting phenotypes are comparable in insect ecdysis. Clearly, ecdysis behaviors in both insects and crustaceans are conserved and extremely ancient. Thus, an important question is: Have the common command peptides viz. ETH and EH and peptide networks involved in ecdysis been conserved with respect to both structure and function during evolution of an essentially aquatic sub-phylum (crustaceans), compared to a primarily terrestrial one (insects)? Certainly, CCAP and bursicon have been conserved regarding structure and function in both, and transcriptomic analysis of neuropeptides have indicated that ETH and EH-like molecules are widely, perhaps universally present in crustaceans. For example, peptides containing the motifs found in insect ETHs, -PRX-NH_2_ where X is I, L, V, or M [36], seem to be universally present in ETH-like molecules of decapod crustaceans, which has thus far been annotated as such [37]. These peptides display almost complete conservation in structure (DAGHFFAETPKHLPRI-NH_2_), and a similar situation exists for EH-like molecules identified in decapods (this study). Nevertheless, very little is currently known regarding the functions of ETH and EH-like peptides in crustaceans, and indeed, whether their presence reflects homologous physiological roles to the well-studied ones of insects.

Here, we explore the functions of ETH and EH in the brachyuran crab *Carcinus* *maenas*, by characterizing the ETH receptor, describing the localization and neuroarchitecture of ETH and EH transcripts and peptides, and measuring in vivo ETH peptide titers in hemolymph. We propose a working model of the crustacean ecdysis cascade founded on temporal release patterns of key molting neurohormones throughout the ecdysis program.

## Results

### Deorphanisation and tissue distribution of the ETH receptor

**Fig. 1:**
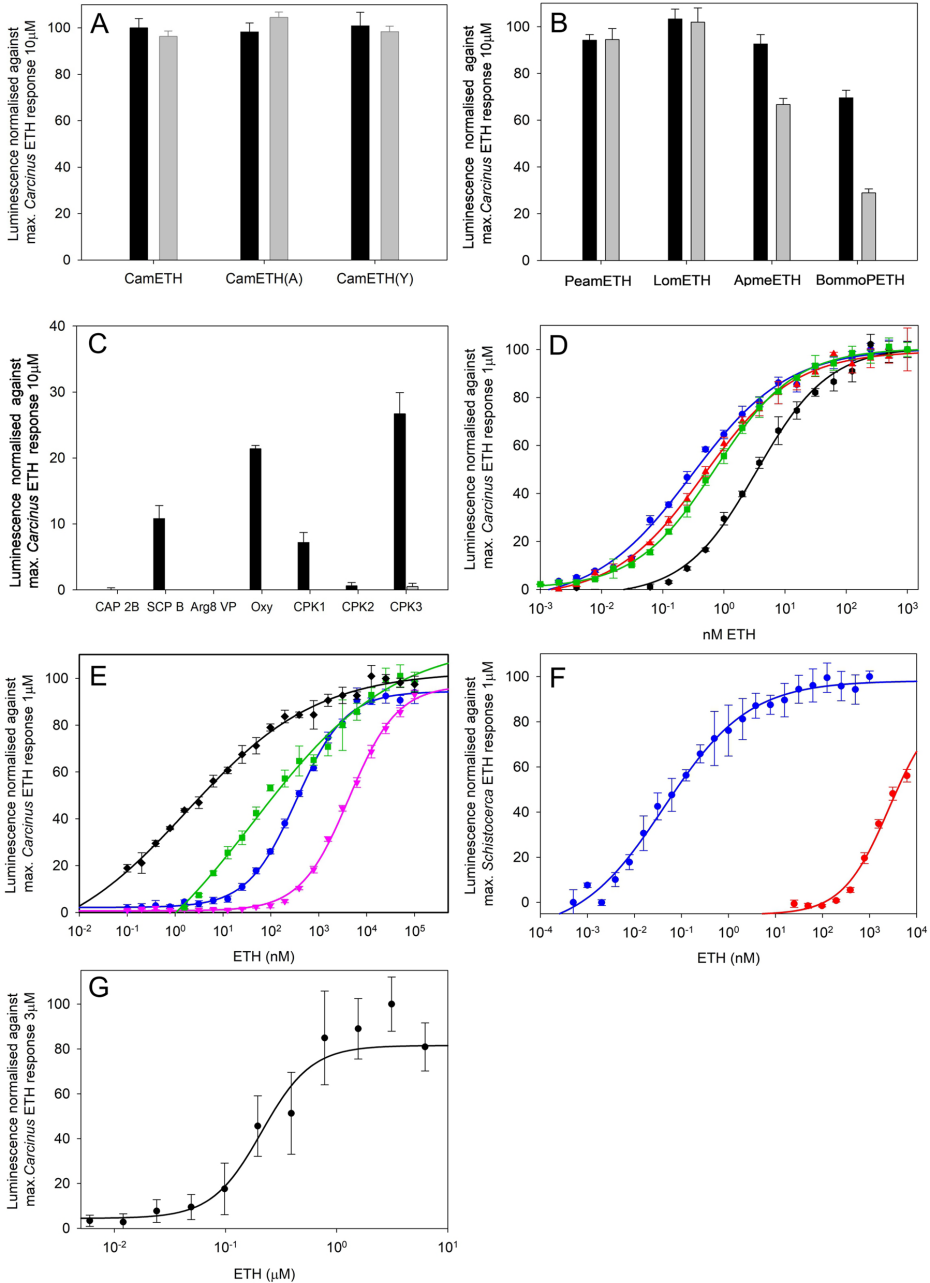
ETH receptor deorphaning. **A** Luminescent responses of CHO-K1-Aeq-Gα16 cells transiently expressing *Cam*ETHR to saturating doses of *Cam*ETHs (*n* = 4 + 1SD). Values normalized against maximum response to 10 µM Cam ETH. Black bars 10 µM, Gray bars 1 µM. Analogs: *Cam*ETH (A); N-terminally extended ETH, *Cam*ETH(Y): Y5 ETH. **B** Luminescent responses of CHO-K1-Aeq-Gα16 cells transiently expressing *Cam*ETHR to Insect ETHs. Black bars 10 µM, Gray bars 1 µM (*n* = 4, Means + 1SD). Responses were normalized against the maximum response obtained for *Cam*ETH (10 µM). Abbreviations: Peam: *Periplaneta americana*, Lom: *Locusta migratoria*, Apme: *Apis mellifera*, Bommo: *Bombyx mori.* **C** Luminescent responses of CHO-K1-Aeq-Gα16 cells transiently expressing *Cam*ETHR to peptides structurally related to ETHs. Black bars 10 µM, Gray bars 1 µM (*N* = 4 Means + 1SD). Abbreviations: CAP2B: cardioactive peptide 2B, SCPB: small cardioactive peptide B, VP: arginine vasopressin, OXY: oxytocin, CPK 1–3 carcipyrokinins 1–3. See Additional file 3: Table 1 for peptide sequences. **D** Dose response curves (*n* = 4. ± 1SD) for bioluminescence of *C. maenas* (*Cam*) ETH and analogs on CHO-K1-Aeq-Gα16 cells transiently expressing *Cam*ETHR. Receptor activation is shown normalized to the highest of *C. maenas* (Cam) ETH value (100%) and corrected for controls (BSA medium) and total bioluminescence following cell lysis. Red: control cell transfections CHO-K1-Aeq-G*q*), *Cam*ETH. Green: *Cam*ETH, Blue: N-terminally extended (A)-*Cam* ETH, Black (Y*5*)- *Cam* ETH. Estimated ED_50s_ 0.4–0.6 nM, Y*5*-*Cam* ETH 3.5 nM. **E** Dose response curves (*n* = 4 ± 1SD) for luminescence of CHO-K1-Aeq-Gα16 cells transiently expressing *Cam*ETHR after activation by insect ETHs. Black: *Locusta migratoria* (ED_50_ *ca*. 3.5 nM), Green: *Periplaneta americana* (ED_50_ *ca*. 110 nM), Blue: *Apis mellifera* (ED_50_ *ca*. 380 nM), Red: *Bombyx mori* PETH (ED_50_ *ca.* 4350 nM). Values normalized to maximum luminescence following addition of 100 nM *Cam*ETH. **F** Luminescent responses of CHO-K1-Aeq-Gα16 cells transiently expressing *SchgrETHR* to *Lom/Scgr*ETH (blue) and *Cam*ETH (red) (*n* = 4 ± 1SD). Values normalized to maximum response to 1 µM *Lom/Scgr*ETH. **G** Dose response curves (*n* = 4 ± 1SD) for luminescence induced by HEK cells co- transfected with a CRE-Luc reporter plasmid (pGL4 CRE-CMVmin-luc) and the *Cam*ETHR construct after a 4-h incubation in substrate (SteadyLite plus ®) as detailed in materials and methods. Receptor activation is shown normalized to the highest luminescence value (4 µM ETH)

The putative *Cam*ETH receptor identified from our *C. maenas* transcriptomes [38] based on similarity to functionally deorphanised insect ETHRs was transiently expressed in CHO-K1-Aeq-Gα16 cells. It was activated by saturating doses of *Cam*ETH, an N-terminally extended analog, and one in which F_5_ had been replaced by a Y residue (Fig. 1A). Similar experiments using insect ETHs showed that while *P. americana* and *L. migratoria* ETHs were similarly activated with respect to the responses obtained with the same doses of *Cam*ETH, *A. mellifera* ETH was less effective, and *B. mori* PETH was even less effective at activating this receptor (Fig. 1B). *Cam* ETHR was minimally activated by 10 µM doses of several peptides displaying the conserved C-terminal PRX-amide motif or vasopressin and oxytocin (Fig. 1C). Dose response curves were constructed for *Cam*ETH, showing ED_50_s of between 0.4 and 0.6 nM for both -Gα16 and –G*q* (control) cells, but replacement of F_5_ with a Y residue resulted in a marked increase in ED_50_ (3.5 nM, Fig. 1D). Dose response curves constructed using insect ETHs on cells expressing the *Cam* ETHR showed that *L. migratoria* ETH was the most effective at activating this receptor (ED_50_ 3,5 nM), followed by *P. americana* ETH (ED_50_ 100 nM), and *A. mellifera* ETH (ED_50_ 380 nM). *B. mori* PETH was scarcely effective at activating this receptor except at µM concentrations (ED_50_ 4.35 µM) (Fig. 1E). Dose response experiments where the *S. gregaria* ETHR was transiently expressed in CHO-K1-Aeq-Gα16 cells showed that while *L. migratoria* ETH (identical to *S. gregaria* ETH) was highly effective in activating this receptor (ED_50_ 0.1 nM), *Cam*ETH was only active at sub µM concentrations (Fig. 1F). Since *Cam*ETH activated its receptor equally in both -Gα16 and -G*q* (control) cells, we used a CRE-Luc reporter plasmid cotransfected with the ETHR construct into HEK cells as detailed earlier. While higher doses (µM) of *Cam*ETH were necessary to show luminescent responses, the dose response curve showed that signaling was also via cAMP (Fig. 1G).

**Fig. 2:**
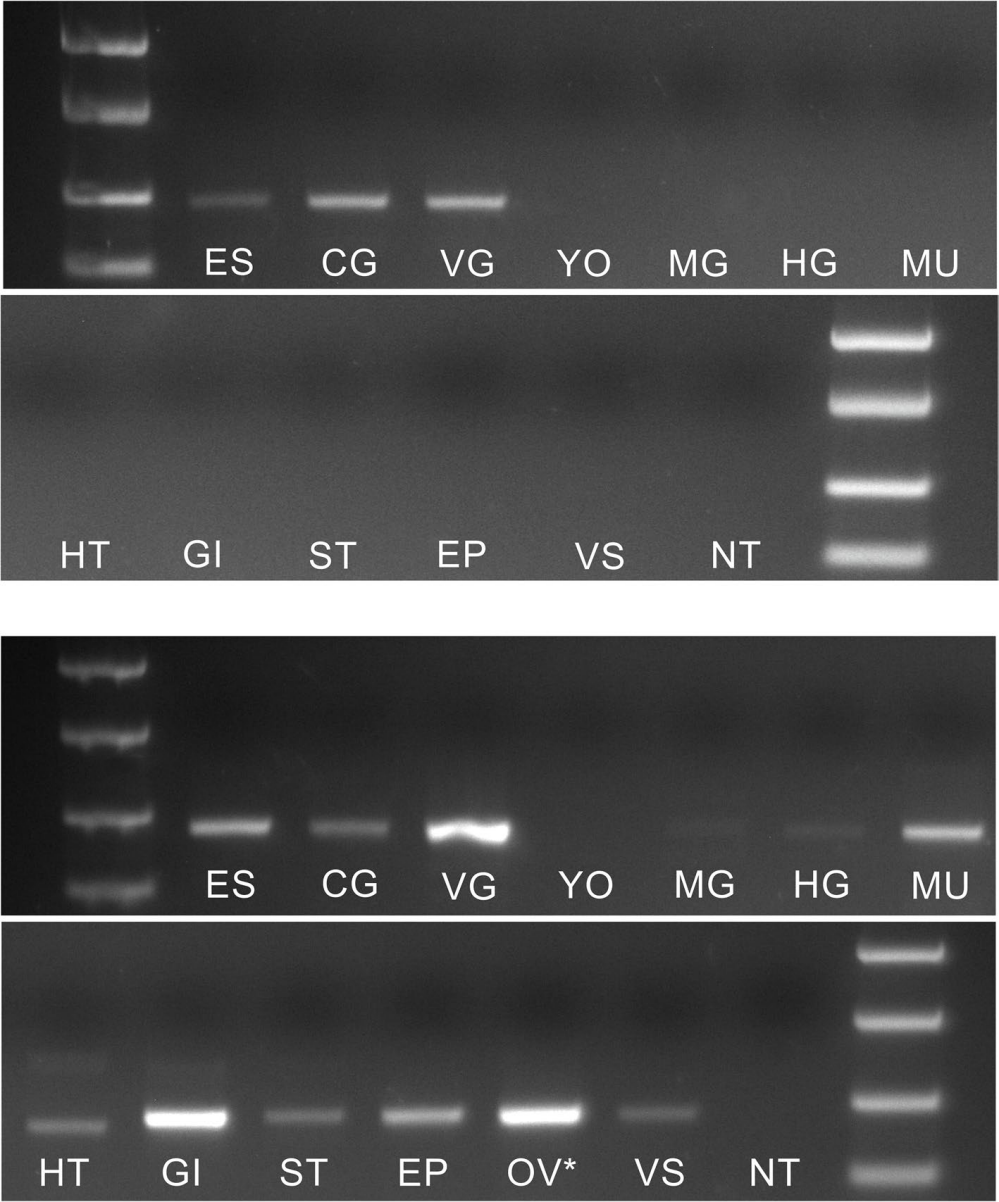
ETH and ETHR tissue expression. End point PCR showing tissue distribution of *ETH* (upper panels) and *ETHR* (lower panels) transcripts in stage D4 crabs. PCR conditions as detailed in the text. Abbreviations: ES, eyestalk; CG, cerebral ganglion; VG, ventral ganglion; YO, Y-organ; MG, midgut gland; HG, hindgut; MU, muscle (dactyl); HT, heart; GI, gill; ST, stomach; EP, epidermis; OV, ovary (stage 4); VS, vas deferens and spermatophores; NT, no template. Asterisk denotes ovarian tissue from intermolt sample. Markers: 2000, 1000, 500, 250 bp

End point PCR of cDNA reverse transcribed mRNA from tissues of (late premolt, stage D4) *C. maenas* showed that several tissues expressed ETHR (Fig. 2). Moderate levels of expression were observed in the nervous system and in particular, gill and ovarian tissues. Low levels of expression were seen in limb muscle, heart, stomach, epidermis, and sperm duct (lower panel). In contrast, ETH expression was limited to the nervous system (upper panel).

### Neuroanatomy of ETH and EH: whole mount immunohistochemistry (IHC) and in situ hybridization (ISH)

#### ETH

An antiserum raised against synthetic ETH was used to determine the morphology of ETH expressing neurons in the central nervous system, together with in situ hybridization to show congruence for immunopositive peptide and transcript neuronal expression. Specificity of labelling was confirmed by preabsorption of a tenfold molar excess of peptide to affinity purified IgG, which almost completely abolished immunolabelling (Additional file 1: Figure S1). Specificity to ETH was further confirmed by HPLC of nervous system extracts followed by quantification by TR-FIA (Additional file 2: Figure S2).

**Fig. 3:**
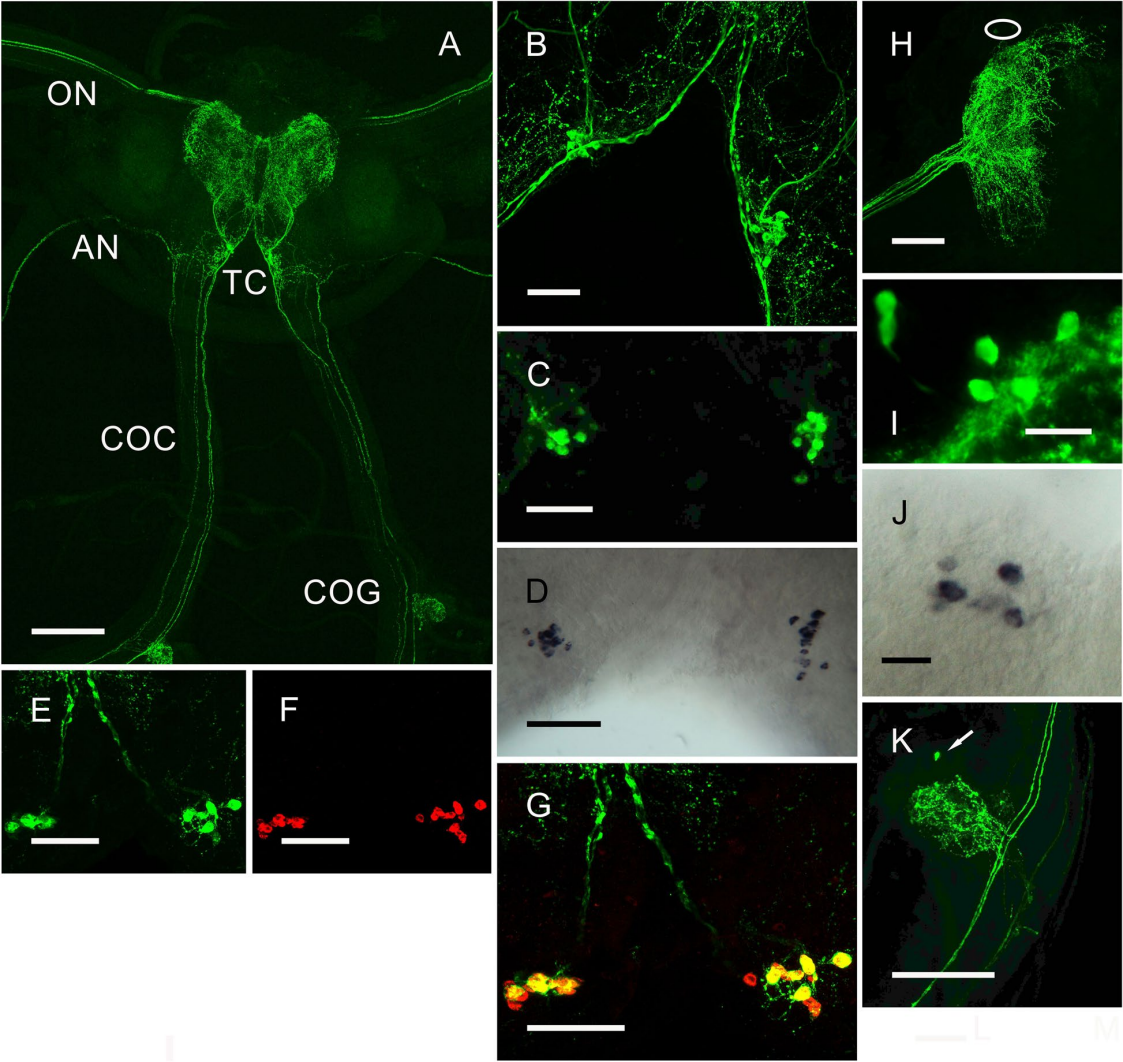
ETH neuroanatomy in the cerebral ganglia and eyestalk: immunohistochemistry and in situ hybridization. Distribution of ETH in the central nervous system of *C. maenas* visualized by confocal IHC (**A**–**C**, **H**, **I**, **K**), mRNA by ISH (**D**, **J**) or HCR-FISH (**E**–**G**). All preparations were from intermolt animals, except where noted. **A** Tiled whole mount image of cerebral ganglion (CG), showing groups of perikarya in the tritocerebrum (TC), projecting descending fibers via the circum-esophageal connectives (COC), with branches in the commissural ganglia (COG). Ascending fibers form complex branching patterns in the CG, and projections to the antennal (AN) and optic nerves (ON). **B** Detail of immunopositive perikarya in the TC. **C** Shallow Z-stack showing the groups of cells shown in **B**. **D** ISH of the groups of perikarya in the TC (Stage D3-4). **E**–**G** Combined IHC and HCR-FISH of the ETH perikarya in the TC. **E** IHC, **F** HCR-FISH, and **G** merged image, showing extensive, essentially complete overlap for transcript and peptide. **H** Ascending fibers from the TC neuron group form extensive branches over the X-organ region of the eyestalk. Highlighted area shows the position of a group of perikarya shown in **I**, which are only visible in stage D4. **J** ISH of the cell group shown in **F**. **K** Branches in the COG of two large descending fibers in the COC. One small cell body (arrow) could be seen in most preparations. Scale bars **A** 200 µm, **B**–**I**, **J** 50 µm, and **K** 100 µm

In the cerebral ganglion, complex neuroarchitecture was observed. In the tritocerebrum between 15 and 20 pairs of neurons (*ca.* 20 µm diameter) projected axons to the optic and antennary nerves and circum-esophageal connectives, with complex branches in the cerebral ganglion (Fig. 3A–C). Similar patterns of mRNA expression were observed for ISH preparations (Fig. 3D) and codetection of peptide and corresponding transcript via HCR-FISH showed complete colocalization (Fig. 3E–G). Notably, the ascending axons projecting to the eyestalk showed complex branching morphology in the X-organ, and during late premolt (D4), 6–8 immunopositive neurons (*ca*. 20 µm diameter) were seen, which were also observed by ISH (Fig. 3H–J). These were not seen at other stages of the molt cycle. In the circum-esophageal connectives, several descending axons were seen, and two prominent axons projected branches into the commissural ganglion (COG), where a single small cell body (*ca*. 15 µm diam.) was observed (Fig. 3K). However, the identity of this could not be confirmed by ISH.

**Fig. 4:**
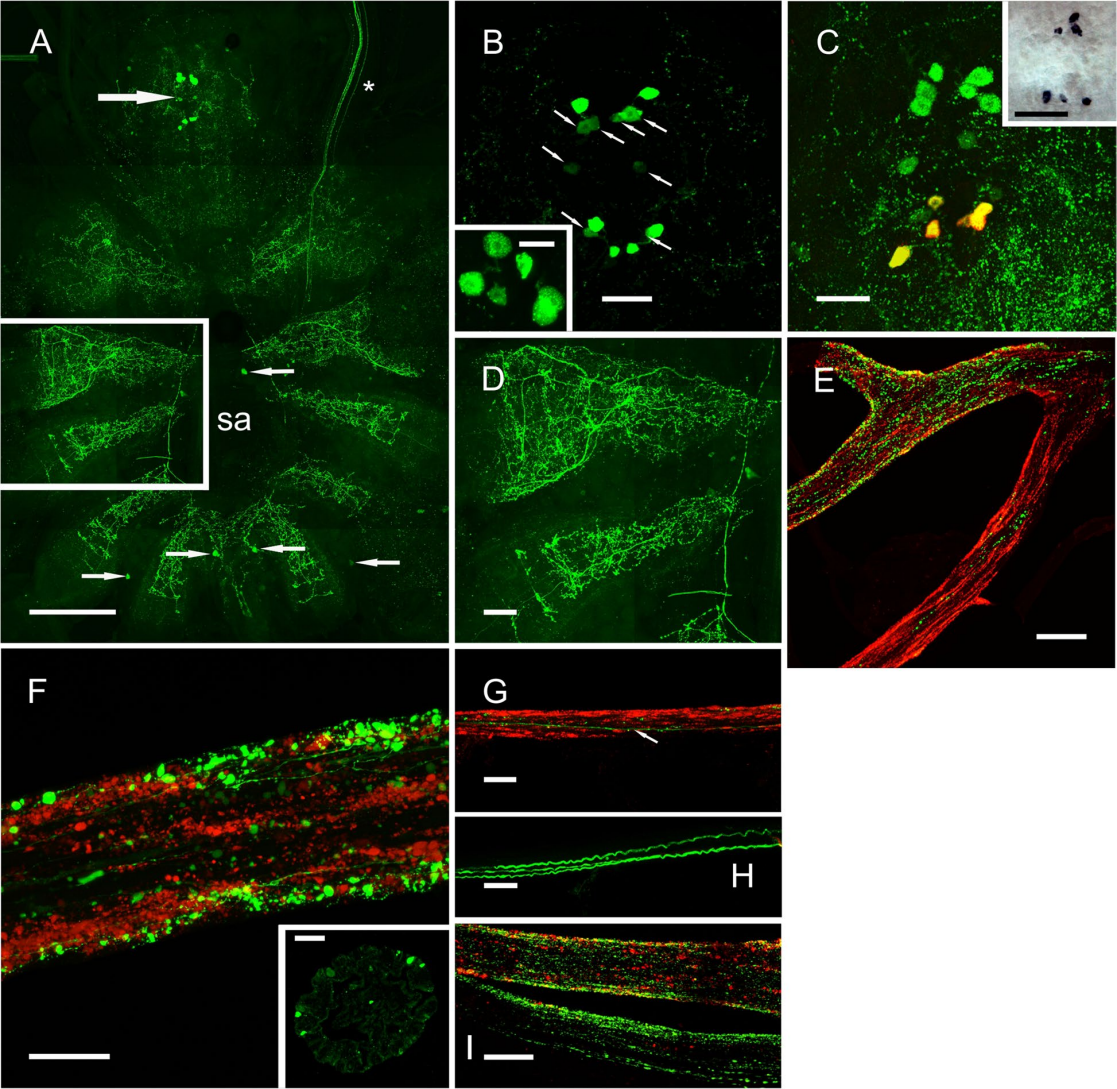
ETH neuroanatomy in the ventral ganglia and pericardial organs: immunohistochemistry and in situ hybridization. **A** Distribution of ETH immunoreactive structures in a whole mount ventral ganglion (VG), produced by a shallow 4 × 3 tile scan Z-stack of the dorsal surface. Large arrow points to a prominent cell group shown in the sub-esophageal ganglion (SOG). Some weakly immunopositive cells are shown (small arrows). Complex branches were seen in each dorsal root of the VG. Boxed region of two roots is shown in **D**. The asterisk indicates a reflexed part of the circum-esophageal connective (COC). sa: sternal artery foramen. **B** ETH immunopositive neurons (*ca*.14 in this preparation) in the sub-esophageal ganglion (SOG). In all preparations, a set of weakly immunopositive cell bodies were seen (arrows). Inset shows details of a neuron group showing punctate cytoplasmic labelling. **C** Combined IHC and HCR-FISH of the SOG ETH neurons. Green labelling shows peptide distribution, yellow labelling, colocalization of transcript and peptide. Inset shows conventional ISH of this cell group (Stage D3-4). **D** Two thoracic roots shown in **A** inset, showing complex branches on the surface of the preparation. **E** Anterior bar of a pericardial organ labelled to show axons, and secretory boutons immunpositive for ETH or bursicon (green and red respectively). In the ventral trunk, very few ETH immunopositive structures were observed. **F** Double labelling of dorsal trunk of the PO showing fibers and secretory boutons immunopositive for ETH (green) and bursicon (red). Inset shows a transverse semithin (1 µm) section of a dorsal trunk showing ETH secretory boutons. **G** Immunolabelled ventral trunk of a PO showing extensive, prominent immunolabelling for bursicon, but sparse labelling, and a single axon profile for ETH (arrow). **H** Segmental nerve showing three prominent axons immunopositive for ETH, but no bursicon labelling. **I** Double labelling of a PO preparation with anti-ETH directly labelled with Alexa 594 (red) and the structurally related Carcipyrokinin-1 (green) showing complete separation of immunopositive structures. Scale bars: **A** 500 µm, **B**–**E**, **G**–**I** 100 µm, **F** 50 µm, **F** inset) 20 um

Whole mount tile scanned Z-stacked preparations of the fused thoracic (TG) and abdominal ganglion (AG) revealed highly complex structures with many fine branches on the surface of the dorsal roots corresponding to thoracic neuromeres 3–8 which innervate the walking legs (Fig. 4A, D). While several weakly immunopositive cells were seen in some preparations (Fig. 4A, small arrows), a prominent group of strongly immunopositive cells (*ca*. 35 µm diam.) were seen in the subesophageal ganglion (Fig. 4A, large arrow). A maximum of 14 cells in anterior and posterior positions were seen, but in many preparations, punctate immunolabelling was observed in some cells (Fig. 4B and inset). An HCR-FISH preparation showed that while all cells contained peptide, expression of transcript appeared to only occur in the posterior cell group (Fig. 4C). However, conventional ISH of stage D4 preparations showed expression in all cells (Fig. 4C inset). While the axonal projections of this cell group could not be seen, labelling patterns of fibers and secretory boutons in the pericardial organs (PO) (Fig. 4E, F) together with three prominent axons in the segmental nerves (Fig. 4H) might represent anatomy relevant to this prominent cell group. Double labelling of the PO showed the peripheral location of secretory boutons (Fig. 4F and insert). Most secretory boutons were found on the anterior and posterior bars, adjacent to the branchiocardiac veins, but few secretory boutons were observed on the ventral trunks, which contained a single immunopositive ETH fiber, in contrast to abundant boutons and fibers immunopositive for bursicon (Fig. 4E, G). Double labelling of PO preparations with Carcipyrokinin-1 and ETH antisera showed that there was no cross reactivity to the two structurally related peptides (Fig. 4I).

#### EH

An antiserum raised against synthetic linear (no disulfide bridges) full length EH was used to determine the morphology of EH expressing neurons in the central nervous system, together with in situ hybridization to show congruence for immunopositive peptide and transcript neuronal expression. Specificity of immunolabelling was confirmed by preabsorption of a tenfold molar excess of peptide to affinity purified IgG, which completely abolished immunolabelling (Additional file 1: Figure S1). Specificity to EH and confirmation of the identity of immunoreactive material was confirmed by HPLC of eyestalk extracts followed by quantification by TR-FIA and electrospray-time of flight mass spectrometry (ES-TOF MS) of the immunopositive fraction confirmed the identity of this material (Additional file 2: Figure S2).

**Fig. 5:**
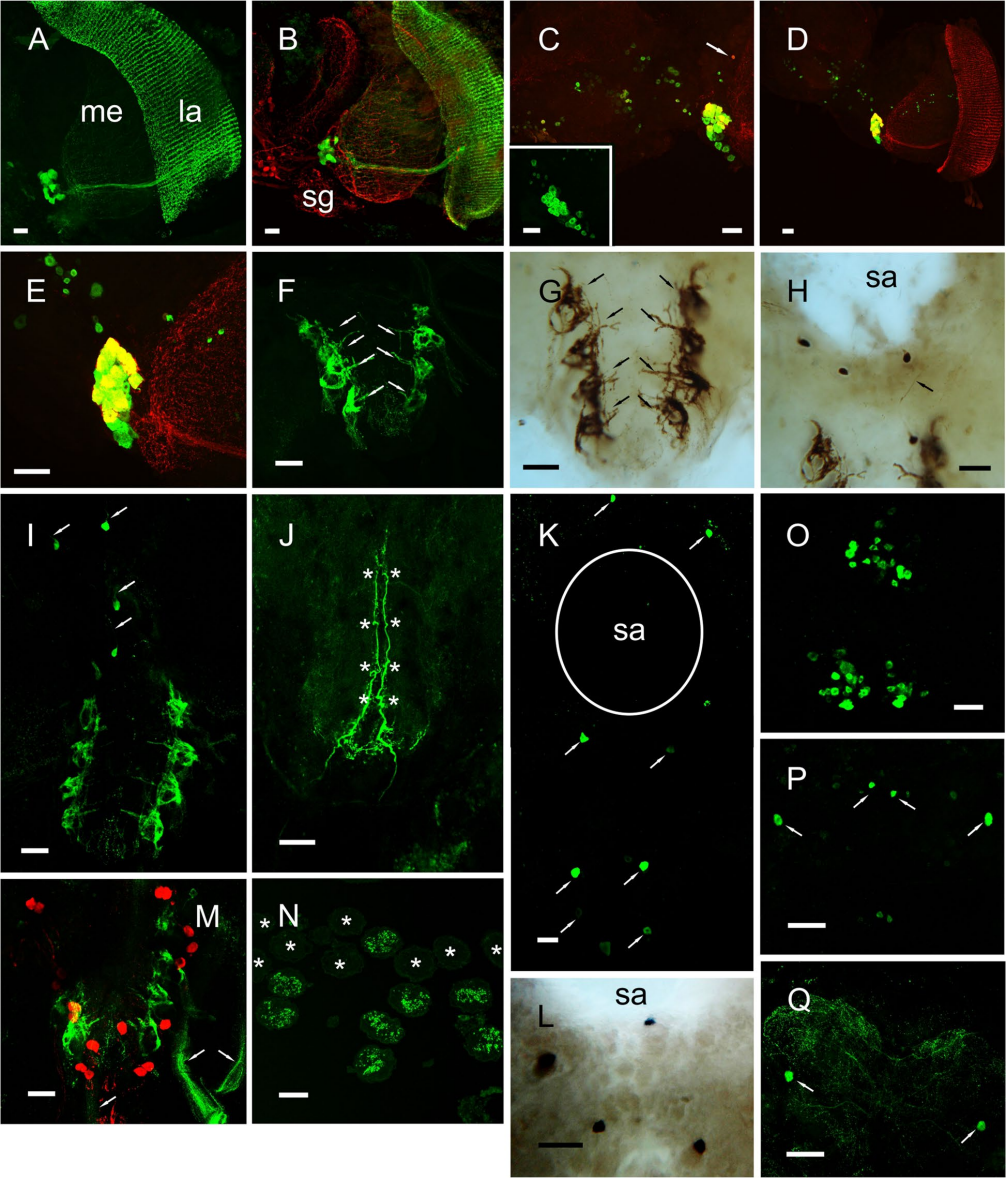
EH neuroanatomy in the CNS: immunohistochemistry and in situ hybridization. Distribution of EH in the central nervous system of *C. maenas* visualized by confocal IHC (**A**, **B**, **F**, **I**, **J**, **M**, **N**, **O**, **Q**), IHC (PAP-DAB) (**G**, **H**), mRNA by ISH (**L**) or HCR-FISH (**C**–**E**, **K**, **O**, **P**). All preparations were from intermolt animals. **A** EH immunoreactive neurons in the eyestalk medulla (me) ca. 15–18 cells (*ca*. 45 µm diam.) projecting axons to the lamina (la). **B** Double labelling of EH (green) and PDH (red) in the eyestalk. Position of the sinus gland (sg) is shown. **C** Combined ISH and HCR-FISH of EH peptide (red) and transcript (green) in the eyestalk. Yellow shows colocalization of both mRNA and peptide in some cells. Approximately 80 small neurons (*ca.*13–16 µm diam.) exhibited only mRNA expression, excepting one cell (arrow), that only expressed peptide. Inset shows expression of EH mRNA in *ca*. 30 cells in the group shown in **A** and **B**. **D** Preparation as described in **C** showing extensive EH immunopositive structures in the eyestalk lamina, and the differential expression of peptide and corresponding transcripts in the large cell group. **E** Detail of cell group shown in **D**. **F** Shallow Z-stack of EH immunopositive structures on the dorsal side of the abdominal ganglion. Four paired axon tracts directing dorsally are indicated (arrows). **G** Wholemount PAP/DAB preparation of EH immunopositive structures in the abdominal ganglion, showing a similar arrangement of structures shown in **F**. **H** Wholemount PAP/DAB preparation showing four cell bodies near the sternal artery foramen (sa). One fine axon appeared to direct anteriorly (arrow). **I** Shallow (dorsal) Z-stack of immunopositive structures in the abdominal ganglion. Four cells (*ca*.25 µm diam.) project fine axons anteriorly (arrows). **J** Shallow (ventral) Z-stack showing a pair of prominent axon tracts. Asterisks indicate four pairs of “kinked” structures. **K**. HCR-FISH (two combined images) showing 4 pairs of cells expressing EH (small arrows). Outline shows position of sternal artery foramen (sa). **L** ISH of four cells adjacent to the sternal artery foramen, corresponding to those seen in **H**, **I** four posterior to the sternal artery foramen in **K**. **M** Double-labelled preparation (Z-stack average) showing bursicon immunopositive cells (red) near the EH immunopositive fiber (green) structures shown in **F**, **G**, and **I**. Abdominal nerves show labelling of many fine fibers in this image (arrows). **N** Transverse 10-µm section of abdominal nerves (8 pairs). Eight nerves (four pairs) showed intense labelling of many small fibers, eight showed no labelling (asterisks). **O** HCR-FISH of cell bodies (*ca*. 25 µm diam.) in the sub-esophageal ganglion. **P** HCR-FISH of cell bodies in the cerebral ganglion. A pair of lateral cells (*ca*.30 µm diam.) and a pair of median cells (*ca*. 20 µm diam.) gave intense signals (small arrows). Several other cells gave weak hybridization signals. **Q** IHC of the cerebral ganglion. A pair of lateral cells corresponding to those seen in **P** were observed. Scale bars: 100 µm

In the eyestalk, a prominent group of 15–18 perikarya (*ca.* 45 µm diam.) in the medulla adjacent to the sinus gland projected axons to the lamina, forming a distinctive, highly complex structure (Fig. 5A) Fine branches were seen in the medulla, which were closely associated, but distinct from, pigment dispersing hormone (PDH) immunopositive structures (Fig. 5B). Combined HCR-FISH and IHC showed the presence of many (*ca*. 80) small neurons (13–16 µm diam.) scattered throughout the medulla, but while these expressed transcripts, EH peptide could not be detected, and the group of large cells expressing EH revealed many more cells that were expressing transcript but not peptide (Fig. 5C–E). In the abdominal ganglion, a complex structure of dorsally positioned fibers, arranged in four pairs, were prominent in confocal and PAP/DAB whole mounts (Fig. 5F–I). Posterior to the sternal artery foramen, 4 neurons were generally seen, which appeared to project fine axons anteriorly, but these could not be traced further. Ventrally directed Z-stacked images revealed two prominent axon tracts, which characteristically exhibited four pairs of “kinks,” which likely corresponded to the fine centrally directed fibers in Fig. 5F, G (arrows). HCR-FISH revealed up to 8 cells expressing transcript (Fig. 5K), but conventional ISH (Fig. 5L) only revealed 4 cells corresponding to those seen by IHC. Double immunolabelling for EH and bursicon showed a close association of bursicon neurons with the four pairs of complex fibers and in most preparations; abdominal nerves contained many fine fibers (Fig. 5M). Transverse sections of the abdominal nerves showed that 8 nerves (corresponding to four pairs) contained the fine fibers, while another 8 did not (Fig. 5N, asterisks). In the sub-esophageal ganglion, many small (*ca.* 25 µm diam.) cells were seen in HCR-FISH preparations, but peptide could not be detected in these (Fig. 5O). In the cerebral ganglion, 2 pairs of median and lateral cells (ca. 20 µm) expressed transcript (Fig. 5P), but only the lateral pair expressed peptide (Fig. 5Q).

### Molt cycle-related changes in ETH expression and circulating hemolymph titers

**Fig. 6:**
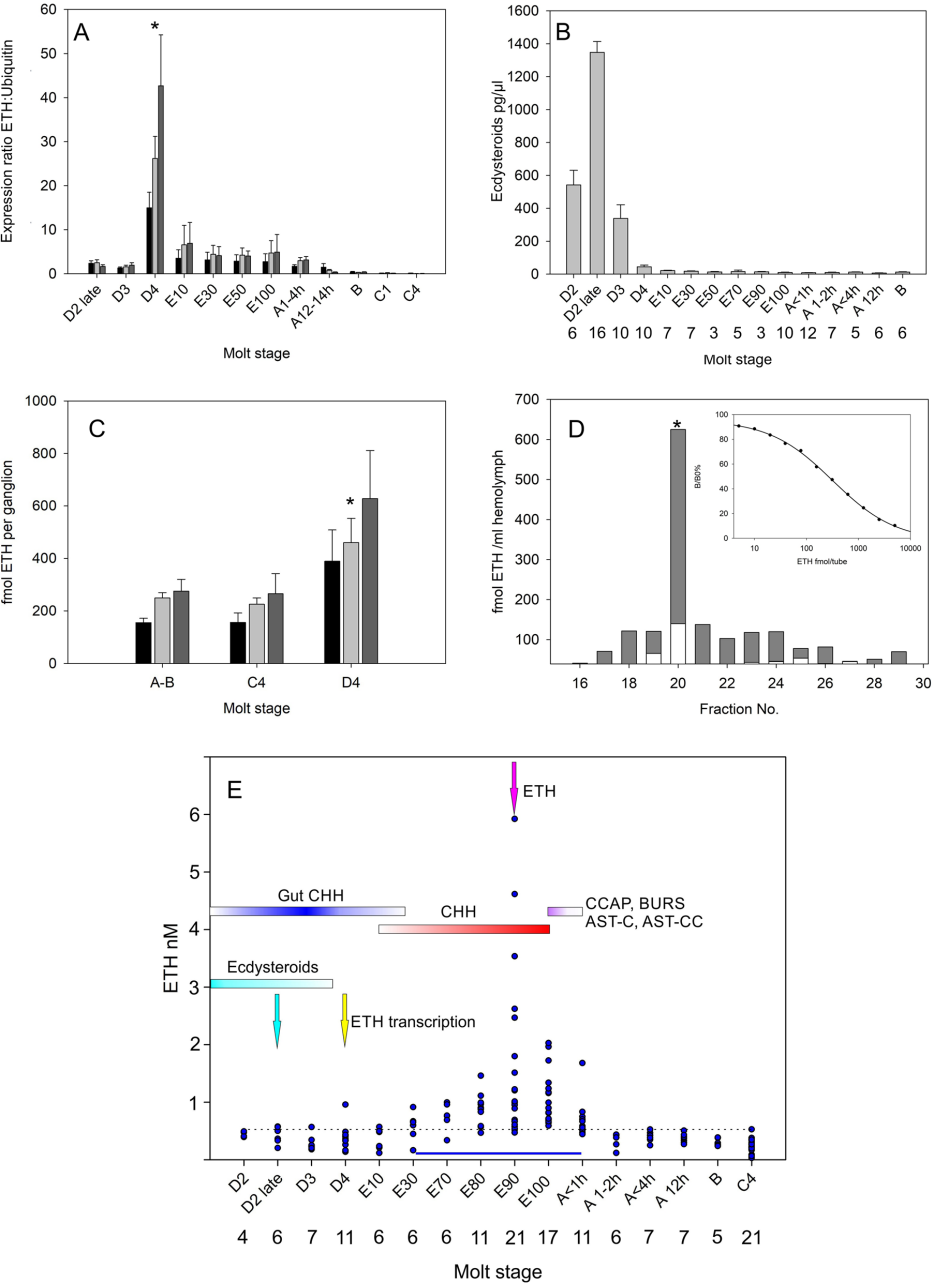
ETH peptide, transcript in tissues, ecdysteroid, and ETH immunoreactive material in purified hemolymph during ecdysis. **A** ddPCR of ETH transcripts in nervous tissues during the molt cycle. Values are normalized against Ubiquitin-conjugating enzyme E2 L3 (UBE2L3) mRNA expression. Means + 1 SEM. *N* = 4–6 at each molt stage and tissue. Asterisk indicates significantly higher levels (Student’s-*T*, *P* < 0.05) of ETH transcripts in stage D4 compared to stage D3, and stage E10 Black bars: eyestalk, gray bars: cerebral ganglion, dark gray bars: ventral ganglion. **B** Ecdysteroid titers in hemolymph extracts during ecdysis. Means + 1SEM. Numbers at each molt stage are shown. **C** ETH levels in Strata-X SPE purified nervous tissue extracts, in postmolt (stage A-B), intermolt (stage C4), and late premolt (stage D4). Means + 1 SEM. *N* = 6–10 at each molt stage and tissues. Asterisk shows significantly higher levels (*P* < 0.05, Student’s-*T*) of ETH in cerebral ganglion tissues during premolt compared to post and intermolt. Black bars: eyestalk, gray bars: cerebral ganglion, dark gray bars: ventral ganglion. **D** HPLC TR-FIA of Strata-X SPE purified 4.5 ml hemolymph samples. Gray bars: stage E90, white bars: stage C4. Asterisk indicates fraction corresponding to injection of 2 pmol synthetic ETH following separation of purified hemolymph samples. Inset shows typical standard curve for this assay. Detection limit ca. 5–10 fmol/well. **E** ETH levels in hemolymph during ecdysis. Dotted line represents highest levels of immunopositive material during intermolt (stage C4). Blue line: region where elevated titers of ETH immunopositive material were significantly different to those at stage C4. Skewed distribution necessitated use of a Generalized Linear Model with a gamma family and log link. Cyan arrow: maximum ecdysteroid titer. Yellow arrow: maximum ETH transcript number in CNS. Red bar: period when CHH released from gut endocrine cells. Blue bar: period when CHH is released into the hemolymph from the gut endocrine cells. Magenta bar: period when CCAP, bursicon (BURS), AST-C/CC are released from the pericardial organs. Shading of bars indicates concentration in hemolymph. Summarized data: CHH from [39], CCAP, bursicon from [40], and AST-C/CC from [41]

Quantitative measurement of ETH mRNA transcript levels in the eyestalk, brain, and ventral ganglion throughout the molt cycle was performed using ddPCR, with a ubiquitin conjugating enzyme E2 L3 normalizer (Fig. 6A). During intermolt expression was scarcely measurable until late premolt, where levels increased dramatically at stage D4, before declining during all stages of the ecdysis program (E10-E100). Following ecdysis, expression then returned to basal levels within 48 h (Stage B). Measurement of circulating levels of ecdysteroids confirmed that the nadir of these corresponded with Stage D4 (Fig. 6B).

Measurement of ETH levels in SPE extracted nervous systems showed a doubling of ETH content in late premolt (D4), but this was highly variable compared to intermolt levels; only cerebral ganglion extracts showed significant increases at this time, compared to intermolt (C4) (Fig. 6C).

Measurement of ETH immunoreactive content of SPE extracted hemolymph samples followed by HPLC, using a competitive TR-FIA for ETH, showed a main peak of immunoreactive material in hemolymph samples from crabs at stage E90 at the retention time of authentic ETH, which was almost absent in intermolt (C4) (Fig. 6D). Nevertheless, a broad distribution of immunoreactive material was seen in premolt samples.

To determine release patterns of ETH, hemolymph samples (*N* = 152) were taken from crabs during precisely staged premolt, ecdysis, post- and intermolt, purified by SPE and measured by TR-FIA (Fig. 6E). Increases in ETH levels were first seen at the start of active ecdysis (E30), peaking *before* emergence from the old cuticle (E90) and subsequently falling upon completion of ecdysis. Within 2 h of ecdysis, levels had fallen to basal levels. Statistical analysis using a GLM with gamma family and log link (to account for skewed distributions) confirmed that ETH levels were significantly raised (*p* < 0.001) relative to basal levels at stage C4 during E30-A < 1 h.

## Discussion

In the last decade, many transcriptomic studies of crustaceans have identified transcripts that bear strong sequence identity to neuropeptides and their presumed GPCRs to those well known to be involved in the insect ecdysis program, for example [38, 42, 43]. Pertinent examples include candidate ETH-like molecules, presumed receptor (ETHR), crustacean cardioactive peptide (CCAP) and receptor (CCAPR), and bursicon and eclosion hormone-like (EH) peptides. Nevertheless, our knowledge of the (integrated) functions of these candidates remains sparse, not least because of the genetic intractability of most crustaceans. In this study, we address this by functionally deorphanizing the ETHR and its cognate ligand (ETH) previously identified in our *C. maenas* transcriptomes [38], identifying neurons and neuroanatomy of ETH and EH candidate peptide and transcripts and measuring transcript expression and peptide levels of ETH during the molt cycle. We link these phenomena to our previous studies on the endocrine cascades during ecdysis [35, 40, 41, 44] in an attempt to unify (or otherwise) neuroendocrine events during the ecdysis program in arthropods.

### Functional deorphanisation of a putative ecdysis triggering hormone receptor

The candidate ETHR (Transcript TR677726 [38]) was activated by the presumed *Cam*ETH. This peptide appears to be identical in all decapod crustaceans [37] and has been given this appellation because of its similarity with the core sequence of many insect ETHs (Additional file 3: Table 1) and [36]. Similarly, the cognate receptor shows strong identity with several insect ETHRs, and in particular, *Schistocerca gregaria* ETHR [45], where a canonical ETHR sequence in TM6 shows high sequence identity (Additional file 4: Figure S3). While the ETH receptor is well known to be expressed as two splice variants (ETHR A, B) in most insects [4, 5, 8, 9, 46] and has been inferred from genomic analysis in several others [36]; for crustaceans, only single ETHRs have been identified, with the exception of the water fleas *Daphnia pulex and D. magna* [36, 47]. A phylogram of selected putative and functionally deorphanised ETHR homologs illustrates the dichotomy between crustacean and insect ETHRs (Additional file 5: Figure S4).

The *C. maenas* ETHR showed quite low or no activation by some members of the large family of PRX-amides [48], for example Cardioactive peptide 2B, Small cardioactive peptide B, and the *Carcinus* pyrokinins 1–3, confirming high specificity to *Carcinus* ETH. When challenged with analogs of *Carcinus* ETH, the canonical FF residues seen in most insect ETHs seemed to be important, since replacement of F_5_ with a Y residue reduced activation by more than fivefold. Extension of the N-terminus with a single A residue did not affect receptor activation. Since the cloned receptor was also activated by ETH in both Gα16 or G*q* Subunit (control cells) and since CRE-Luc plasmids co-transfected with the ETHR expressing plasmid used in the aequorin assays showed signaling via cAMP, it seems likely that dual Ca^2+^ and cAMP signaling pathways are activated after ligand/receptor binding, as has also been shown for the ETHR of *S. gregaria* [45]*.* The sensitivity response of the *C. maenas* ETH (EC_50_ 0.4–0.6 nM) was very similar to that of *Locusta* (= *Schistocerca*) ETH (EC_50_ 3.5 nM). Curiously, in the reverse experimental scenario, while the *Schistocerca* ETHR was activated by very low doses of its homologous ligand (EC_50_ 0.1 nM), the heterologous ligand (*Carcinus* ETH) showed a vividly asymmetrical response; greater than 100 nM doses were needed to activate the *Schistocerca* ETH receptor. When the cloned ETH receptor was challenged with some insect ETH/PETH peptides, the *Locusta* (= *Schistocerca*) ETH showed very similar dose response curves to *Carcinus* ETH, which probably reflects the somewhat higher sequence identity than with other insect peptides. Indeed, the most structurally divergent peptide (*Bombyx mori* PETH) only activated the *Carcinus* receptor at high nanomolar concentration while *Periplaneta* and *Apis* ETHs have intermediate responses (EC_50_ 110 and 380 nM, respectively). If these experiments are considered in the light of insect and crustacean ETH and ETHR identity and evolution, it seems likely that we have confirmed the cognate ligand receptor pair.

Regarding other proposed crustacean (decapod) ETHRs, to date, only one, from the mud crab *Scylla paramamosain*, has been functionally deorphanized [49] using a cotransfected CRE-Luc reporter and ETHR construct transiently expressed in HEK 293 T cells. The EC_50_ (75 nM) was much higher than in our aequorin-based reporter system (0.4–0.6 nM). The sequence of this receptor, except for a unique N-terminal, is almost identical with the *Carcinus* receptor; if both peptides have start methionines corresponding to Met^6^ of the *Carcinus* protein, only 11/420 residues differ. The only other GPCR annotated as a putative ETHR that we are aware of is from *Macrobrachium nipponense* [50] which has a much lower sequence similarity with these ETHRs. Some other putative ETHRs from *Procambarus clarkii*, *Homarus americanus*, and *Penaeus monodon* have been identified in genomic assemblies but have not been classed as such and cluster separately from other crustacean ETHRs (Additional file 5: Figure S4).

Regarding tissues expressing ETH, RT-PCR clearly demonstrated that its expression was limited to the CNS, whereas ETHR is expressed not only in the CNS but in several other tissues, particularly gill and ovarian tissues. The latter was also found in *S. paramamosain* [49]. Short- (12 h) and long-term (3 × over 16 days) injections of ETH increased the vitellogenic index and expression of ETHR in these crabs. In that study, incubation of ETH (10^−8^ M for 13 h) also increased expression of the Halloween genes (*Disembodied* and *Shadow*) involved in ecdysteroid biosynthesis by the Y-organs and resulted in increased 20-hydroxyecdysone levels in these animals. Nevertheless, we have found in all our studies on *Carcinus* GPCRs that the GPCR mRNAs we have functionally deorphaned are invariably highly expressed in ovarian tissues, including those for corazonin, red pigment concentrating hormone diuretic hormone-31, pigment dispersing hormones [51–53] and CCAP (unpublished) which might suggest that such expression may reflect an abundance of many maternally derived mRNAs in the oocytes [54]. Furthermore, we did not observe any detectable ETHR expression in Y-organs. Clearly, these differences in two very closely related portunid crabs need further investigation before any firm conclusions regarding the action of ETH in reproduction and molting can be drawn.

### Expression of ETH in the nervous system

The limited expression of ETH mRNA to the nervous system prompted us to investigate this further by immunohistochemistry and in situ hybridization, particularly given that ETH expression in all insects is exclusive to the Inka cells (or homologs) adjacent to the tracheal system [10, 12, 36]. Wholemount confocal IHC revealed a complex neuroarchitecture throughout the nervous system showing extensive branching and secretory boutons which indicate neuromodulatory/transmitter and neurohormonal roles. Conventional ISH showed colocalization of mRNA and peptide, and to confirm this, simultaneous HCR-FISH and IHC and conventional ISH were performed. While these experiments showed complete colocalization in the prominent group of ETH perikarya in the tritocerebrum by HCR-FISH (Fig. 3E–G), we observed differential expression in the cell group in the SOG where ETH mRNA appeared to be absent in the anterior cell group. However, in samples taken during late premolt, both cell groups could be detected via conventional ISH, notwithstanding some inevitable cell loss during desheathing (Fig. 4C insert). A notable feature of these perikarya was the variable number of cells that could be observed via IHC. In general, 14 cells were observed, but invariably some labelling was faint and punctate (Fig. 4B). In the eyestalk, 6 perikarya adjacent to the X-organ of the medulla were only observed in late premolt, by both IHC and ISH, which might suggest some changes in expression during the molt cycle, as will be alluded to later.

The pericardial organs (PO) exhibited intense immunolabelling indicative of a neurohormonal role for ETH since axons, varicosities, and secretory boutons on the surface of the PO were prominent. Labelling was distinct from the bursicon/CCAP/AST-C neurosecretory system, where bursicon immunopositive structures also contain the latter two peptides [40, 55]. A distinction between fibers and secretory boutons immunopositive for the structurally related carcipyrokinin-1 and ETH (PRL/I amides) could also be made, further indicating that immunolabelling for ETH was highly specific. A notable feature of ETH labelling in the PO was that it was restricted to regions on the anterior and posterior bars directly adjacent to the branchiocardiac veins but was almost absent in the ventral trunks. This contrasted with the rather uniform distribution of the bursicon/CCAP/AST-C axons and secretory boutons throughout the PO trunks and bars. Regarding the neuronal input of ETH to the PO, little can be said with certainty. A single segmental nerve contained three prominent axon bundles, without bursicon labelling. It thus seems likely that input was from the cell groups in the SOG, but unfortunately, their fine axons could not be traced.

### Expression of an EH-like peptide in the nervous system

Eclosion hormone-like neuropeptides are widespread, perhaps universal in crustaceans, as evidenced by their annotation in most, if not all crustacean transcriptomes. Generally, two are present in decapods [38], but one (EH-2) is expressed at low levels in many somatic tissues in *Carcinus* [38, 42] and bears limited sequence similarity to that first described in the silkworm, *Bombyx mori* so is not considered to be a *bona fide* neuropeptide. The other EH-like peptide (EH-1) is exclusively expressed in the CNS and in particularly high levels in the eyestalk compared to lower expression in the brain and ventral ganglia [38]. Sequences and phylogram of selected EH-1-like molecules show that their sequences have been extraordinarily highly conserved in decapod crustaceans (Additional file 6: Figure S5).

We used an antiserum raised against synthetic (56 residue linear peptide) *Carcinus* EH which showed high specificity in preabsorption controls. HPLC TR-FIA, mass spectrometry (ES-TOF MS) confirmed the identity of this molecule (with three disulfide bridges) by mass in the fraction containing EH immunoreactive material, further verifying the specificity of the antiserum (Additional files 1, 2: Figures S1, S2). Wholemount IHC showed a complex neural architecture of perikarya adjacent to the SG (in close association with, but separate from, PDH expressing neurons) directing axons to the lamina, where many complex branches were seen throughout this tissue. HCR-FISH confirmed the identity of these cells, revealing that many more exhibited mRNA but no detectable peptide expression in this cell group, and the presence of many (*ca*. 80) small neurons expressing mRNA but no detectable peptide. This may reflect the exquisite sensitivity of HCR-FISH compared to IHC. The architecture of these EH expressing neurons was strikingly like the recently discovered non-canonical EH expressing dorsolateral (D_l_) cells in the *Drosophila* brain [55]. Loss of function of these and the well-known EH containing ventromedial (Vm) neurons results in severe wing expansion defects and very high ecdysis mortality in pharate adults compared to the rather less severe defects of Vm loss of function flies. Furthermore, these neurons do not express either isoform of ETHR implying yet unknown signaling mechanisms. It has been suggested that release of EH from branches of these cells might also modulate visual processing, since it has long been known that bright light gates eclosion in flies [56]. Regarding the functions of the eyestalk EH neural system in crabs, little can be said with certainty. Eyestalk ablation during premolt invariably leads to very high mortality, but this is a very crude tool! However, there is anecdotal evidence for a circadian component of ecdysis in *Carcinus*, with the majority of ecdysis taking place during darkness, notwithstanding firm experimental evidence for a circatidal and semilunar rhythm of ecdysis during the metamorphic molt (megalopae to first crab) in *Carcinus* [57].

In the brain of *Carcinus*, IHC and ISH revealed only a few neurons. These did not project posteriorly, as is characteristic in many insects. However, in the thoracic ganglion, 2 or sometimes 3 pairs of (dorsal) neurons, close to the sternal artery foramen, were detected by IHC, and up to 4 pairs by HCR-FISH. While the very fine axons projecting from these could not be easily traced, immediately posterior to these cells, a large complex structure on the dorsal aspect of the abdominal ganglion was prominent. This consisted of four pairs of dense fiber bundles with a characteristic “blebbed” morphology, each pair of which likely corresponded to abdominal neuromeres 1–4. This structure was reminiscent of a neurohemal tissue and axon tracts on the ventral aspect directed fibers to this structure, which presumably arose from the nearby EH perikarya. However, firm evidence to verify the neurohemal nature of this structure should be made by ultrastructural studies which are now timely, to show whether there is association with blood vessels in the neuropile. Additionally, four of the eight pairs of abdominal nerves contained many fine immunopositive fibers projecting from this structure. Interestingly, this putative neurohemal tissue was near the bursicon, CCAP, and AST-C abdominal neurons. To our knowledge (and rather surprisingly), this structure has not been previously observed, and this now needs further investigation.

EH knockdown by dsRNAi has been reported in the mud crab *S. paramamosain* where crabs injected at the beginning of premolt (D0) died during mid premolt (before stage D2). Similarly, RNAi constructs for ETH and CCAP resulted in somewhat similar mortality patterns [58], and complete ecdysis failure has also been reported in insects subjected to dsRNAi of these peptides, for example, *Tribolium castaneum* [9]. While we considered dsRNAi injection strategies, the extended premolt period in *Carcinus* of a similar size to *S. paramamosain* would have made a strategy involving single injections impractical and unlikely to succeed. Additionally, as alluded to later, while EH transcription increases gradually during premolt, ETH is only transcribed for a brief (one day) period during late premolt (Stage D3-4 [38] and this study). Thus, experimental determination of the optimal time frame in which successful knockdown could be achieved would be technically challenging.

### Molt cycle-related changes in ETH expression and circulating hemolymph titers

In a previous study [38], we found that ETH transcript level in the CNS increased dramatically during late premolt (Stage D3-4), before returning to low levels after ecdysis. Refining these experiments by ddPCR to determine tissue and stage specific expression in the CNS showed that transcript levels in the eyestalk, brain and ventral ganglion increased dramatically during stage D4, which occurred about a day before the initiation of ecdysis, when levels fell to low values during ecdysis, declining still further in post and intermolt. In *Manduca*, high levels of ecdysteroids preceded elevated expression of ETH and ETHR, but competence of the Inka cells to release ETH only occurs after ecdysteroids have fallen to low levels [19]. In *Carcinus*, the peak in circulating ecdysteroid levels occurs in late D2, a few days before entering stage D4 when transcription of ETH increases, which clearly differs from the situation in *Manduca.* However, despite the large changes in transcript level, we did not see a correspondingly large increase in peptide levels in the CNS. Here, levels of ETH increased 2- to threefold in stage D4 relative to intermolt, but they were extremely variable at this time, while for CCAP and bursicon, levels in the CNS remain unchanged in premolt, compared to intermolt [40].

Measurement of ETH immunoreactive material in the hemolymph showed that this material co-chromatographed with authentic ETH, although there was some non-specific activity in other fractions, thus the values obtained are likely to be overestimated. Because of the non-normal skewed distribution of values, statistical analysis was via a GLM. This showed that ETH levels were significantly higher (than intermolt levels) during the entire “active” ecdysis period (E30-E100) and < 1 h after completion of ecdysis. In *Manduca*, levels of ETH rise during pre-ecdysis from 5 nM to *ca*. 30 nM about 20 min later, declining to around 15 nM by the onset of ecdysis [2]. Thus, ETH is released later in the ecdysis program in *Carcinus* than in *Manduca*. While the source of circulating ETH in insects are the Inka cells (or homologs), and while considerable reduction in peptide content is observed following peptide release [2], it is not yet clear whether a similar reduction in ETH content is seen in the PO after release just prior to completion of ecdysis. Since the distribution of ETH immunoreactive material was highly localized to the regions adjacent to the branchiocardiac veins, dissection of complete PO with these areas intact was problematic in pre- and post-molt animals when the PO are almost transparent and extremely fragile. In intermolt crabs, complete successful dissection of POs gave ETH concentrations of around 20 pmol, while incomplete ones were much lower (1 pmol). This variability was not seen for bursicon [40] or CCAP [35], and these peptides did not exhibit spatial variation in content in the PO. However, a total (paired) PO ETH content of 40 pmol would easily account for the low nanomolar levels of ETH seen during the period of release. In view of the release patterns of ETH late in the ecdysis program, we did not attempt peptide injection, since this would have had immediately terminated ecdysis due to uncontrolled hemolymph loss. Injection of ETH into intermolt juvenile crayfish *Cherax quadricarinatus* (10–40 nmol), apparently doubles molt cycle length compared to water injected controls, and a behavioral phenotype involving rapid eye twitching occurred in these animals [37] which we have never observed during ecdysis of crayfish or crabs [35]. Clearly, the increase in molt cycle length cannot be attributable with a physiologically relevant response and given the extremely short half-time of neurohormones in circulation, these results are difficult to explain.

Considering our previous studies showing release patterns of CHH, CCAP, bursicon, AST-C [35, 39–41] we can reconstruct the following series of events during the ecdysis program of *Carcinus*, which are diagrammatically represented in Fig. 6. Following the peak in hemolymph ecdysteroids, CHH is expressed in gut endocrine cells during stage D3. As ecdysteroid levels reach a nadir in stage D3-4, ETH transcription rapidly increases (but resulting in relatively modest increases in ETH peptide levels in the CNS). At the beginning of “active” ecdysis (E10-20), CHH from the gut endocrine cells begins to be released into the hemolymph, reaching levels of around 500 pM by completion of ecdysis, then falling rapidly to low levels (50 pM within 1 h of ecdysis). This release results in notable dipsogenesis and swelling necessary for emergence from the old shell and increases to final postmolt size and complete depletion of CHH in the gut endocrine cells. At the start of active ecdysis ETH begins to be released from the PO, reaching a peak (ca ≥ 2 nM at E90), a few minutes before complete emergence from the old cuticle (E100), then declines back to low levels (< 500 pM) within 1–2 h of completion of ecdysis. CCAP and AST-C are co-released from the PO, beginning at E70-90, and the peak of these and bursicon occurs at completion of emergence. Peak levels are CCAP and AST-C: 800 pM–1 nM, bursicon: *ca*. 4 nM. Since EH release causes increases in cGMP in CCAP neurons in *Manduca* [23, 24], and in view of the close association of the presumed EH neurohemal tissue in abdominal neuromeres 1–4, a speculative scenario might be one in which ETH released into the hemolymph causes release of EH from the neurohemal area in the abdominal ganglion, which then acts upon the adjacent neurons resulting in release of CCAP, bursicon, AST-C. Alternatively, ETH could be directly effecting release of neurohormones at ecdysis, similar to the scenario proposed for the *Drosophila* ecdysis model [8, 10].

## Conclusions

In summary, our data provide convincing evidence for the existence of an ETH/ETHR system in crustaceans, and this is strengthened by demonstration that insect ETHs activate the crab receptor and that related peptides do not. In contrast to insects, ETH is only expressed in neurons, and the neuroanatomy of this neuroendocrine system suggests both neuromodulatory/neurotransmitter functions, as well as an important neuroendocrine role. The eclosion hormone-like peptide has been shown to be present in a new neuroendocrine system, quite different from that seen in insects. When ETH release profiles were measured during the ecdysis program, this occurs rather later than those seen in insects, but is closely associated with dynamic, rapid release patterns of other neurohormones in the “ecdysis cascade.” Given the common origin of arthropods, from a presumed crustacean-like ancestor, it is apparent that while commonalities in the neuroendocrine control of ecdysis occur, there has been considerable diversification in function over evolutionary time.

## Methods

### Animals and tissue collection

Specimens of mature green shore crabs *C. maenas* were collected using baited traps or by hand collection from the Menai Strait, UK. Crabs were maintained in a recirculating seawater system at ambient temperature and photoperiod and fed with fish. For accurate molt staging of premolt and postmolt crabs, specimens were housed individually, and molt staged [59]. For fine scale temporal staging of crabs undergoing ecdysis, behavioral and morphological criteria detailed in [35] were used. Hemolymph samples (1–2 ml) were taken from the hypobranchial sinus, and rapidly frozen in liquid nitrogen. Nervous systems and a selection of tissues were dissected in ice cold *Carcinus* saline [60] following deep anesthesia on ice (1 h). Subsequently, nervous systems were snap frozen in liquid nitrogen for subsequent peptide quantification, RNA work or processed for immunohistochemistry (IHC) by overnight fixation (4 °C) in Stefanini’s fixative [61], or for in situ hybridization (ISH) in 4% paraformaldehyde (PFA) in phosphate buffered saline (PBS), overnight, 4 °C) followed by dehydration in a methanol series.

### Immunohistochemistry and in situ hybridization

Fixed nervous systems were processed for whole mount IHC as previously described [40]. Abdominal ganglia were fixed in Stefanini’s fixative, dehydrated and embedded in Paraplast® for sectioning (10 µm). Pericardial organs embedded in Spurr’s resin were prepared for semithin (1 µm) sectioning as previously described [44]. Antisera were raised in rabbits against candidate ETH, EH, and Carcipyrokinin-1 that were commercially produced as synthetic peptides (Genecust, Dudelange, Luxembourg). Antiserum production (Davids Biotechnologie, Ulm Germany) used ETH or carcipyrokinin-1 N-terminally conjugated to bovine thyroglobulin using 1-ethyl-3-(3-dimethyl-amino-propyl) carbodiimide, or for EH, unconjugated linear full length 56 residue peptide (lacking disulfide bridges). All antisera were supplied affinity purified (binding and elution of antibody from immobilized peptide). An antiserum raised in guinea pig against native *C. maenas* bursicon (Code: SYC392), was as previously described [44]. Pre-absorption specificity controls were performed using tenfold molar excess of ligand to antisera (IgG content).

Whole mount IHC on fixed nervous systems was performed as previously described [44]. Primary antiserum dilutions were 1: 2000 (dilution from stock affinity purified antisera, containing 1.2–1.4 mg/ml IgG). Secondary antisera were Alexa Fluor®488 goat anti-rabbit, Alexa Fluor® 594 goat anti-guinea pig,1:750 (Life Technologies Corp., Eugene, Oregon, USA). Preparations were mounted on cavity microscope slides with Vectashield (Vectorlabs, Newark, CA, USA), coverslipped and sealed with nail varnish. Confocal images were collected and Z-stacked (ca. 25–30 images at 5–10-µm intervals) on a Zeiss LSM 710 confocal microscope equipped with Zen 10 software (Carl Zeiss AG, Jena, Germany).

In situ hybridization was done using digoxygenin-labelled antisense riboprobes as previously described [62]. For conventional ISH, probe synthesis was performed using primers (Additional file 3: Table 1). After desheathing in 50% glycerol/PBS, and mounting as detailed above, images were stacked and assembled using Helicon Focus 6 (HeliconSoft, Kharkiv, Ukraine).

HCR-FISH was performed on wholemount formalin fixed tissues, based on the methodology described [63], adapted from [64]. Custom probes, hairpin amplifiers (with Alexa Fluors®), hybridization and amplification buffers were purchased from Molecular instruments (Los Angeles, CA, USA). Probes were custom designed and synthesized against *C. maenas* mRNA sequences and assigned unique identifier codes by this supplier.

For HCR-FISH, tissues were dissected under nuclease-free, ice-chilled *Carcinus* saline and immediately fixed in 4% PFA, PBS for 16 h at room temperature (RT). Following fixation, tissues were washed in PTW (PBS containing 0.1% Tween-20), 3 × 10 min, dehydrated in a PTW/methanol series (33, 66, 100% methanol) and stored at − 20 °C until use. Tissues were rehydrated though the same PTW/MeOH series, washed in PTW (3 × 10 min), before permeabilization (1.0% SDS, 0.5% Tween 20, 50 mM Tris–HCl (pH 7.5), 1.0 mM EDTA (pH 8.0), 150 mM NaCl) for 30 min at RT. Tissues were subsequently prehybridized in pre-warmed hybridization buffer (37 °C, 1 h). ETH and EH probes (1 mM, Codes RTE 282, RTA 562, respectively) were diluted to 10 nM in hybridization buffer) and hybridized at 37 °C for 48 h. Preparations were then washed in prewarmed wash buffer (4 × 15 min.), followed by 3 × 5 min washes in 5 × SSC, 0.1%Tween-20 (SSCT). Tissues were incubated in amplification buffer for 1 h, RT, followed by hairpins in amplification buffer at RT for 48 h. For ETH hairpin set B1 h1, h2 with Alexa 647 were used, and for EH, hairpin set B2 h1, h2 with Alexa 488 were used. All hairpins (3 mM) were denatured (95 °C, 5 min) and cooled before dilution in amplification buffer to a final concentration of 96 nM.

Following amplification, preparations were washed in 5 × SSCT (2 × 15 min, 2 × 30 min.) before clearing in Vectashield Plus (4 °C, 16 h), mounted and coverslipped as described. Confocal microscopy was performed on a Leica SP8 Super resolution confocal microscope (Leica Microsystems, Milton Keynes, UK). Z stacks were assembled using Leica LAX proprietary software or a Zeiss LSM 710 confocal microscope equipped with Zen 10 software. Images were cropped, resized, and adjusted for brightness and contrast using Adobe Photoshop 2023 and CorelDraw 2014.

For combined HCR-FISH and IHC, preparations were simultaneously processed by adding the antiserum at the amplification step followed by extensive washing in 5 × SSCT (overnight, RT). For ETH transcript and peptide detection (hairpins with Alexa 647), a second antibody Alexa 488 goat anti-rabbit (1:750 RT, 24 h) was used. For EH transcript and peptide detection (hairpins with Alexa 488) Alexa 594 goat anti-rabbit second antibody (1:750 RT, 24 h) were used. For combined EH transcript and bursicon peptide detection, the second antibody was Alexa 594 goat anti-guinea pig. (1:750, 48 h).

### Immunoassays

a) ETH Time resolved-fluoroimmunoassay (TR-FIA). This assay was performed in a competitive format in Goat anti-rabbit coated 96 well microtiter plates (Biomat, Trento, Italy). Standard ETH concentrations were 2500–5 fmol/50 µl, N-terminally biotinylated ETH, 100 fmol/50 µl, affinity purified anti-ETH IgG, 50 ng/50 µl. All were dissolved in assay buffer (50 mM Na_2_HPO_4_ pH7.5, 0.15 M NaCl, 10 mM MgCl_2_, 0.05% casein). Hemolymph samples (1 ml) were gently thawed (to minimize possible degradation of peptides in hemolymph), centrifuged (3300 G, 4 °C, 5 min), diluted with an equal volume of water and purified on Strata-X columns (33 µm polymeric reverse phase 200 mg) by elution on a vacuum manifold (Phenomenex, Macclesfield, UK). After washing with 5 ml water, peptides were eluted with 3 ml 40% isopropanol and dried by vacuum centrifugation and reconstituted in 100 µl assay buffer. For nervous system tissues, samples were disrupted by sonication in 200 µl ice-cold 2 M acetic acid, diluted to 2 ml in water before purification. Under these conditions, recoveries were around 90%. Standard and unknowns (final volumes: 150 µl/well) were assayed in duplicate. Detection limit 5–10 fmol ETH per well. Following overnight incubation, plates were washed (5 ×) in DELFIA buffer (PerkinElmer, Waltham, MA, USA), incubated for 2 h RT in Europium-labelled streptavidin (PerkinElmer) diluted to 100 ng/ml in proprietary assay buffer (Perkin Elmer), 150 µl/well. After washing (5 ×), 100 µl/well enhancement solution (Perkin Elmer) was added followed by orbital shaking (2 min). Time-resolved fluorescence was then measured on a Berthold Mithras LB 940 instrument (Berthold Technologies GmbH, Bad Wildbad, Germany) and data reduction and analysis performed using MikroWin v5.18 (Mikrotek Laborsysteme GmbH, Overrath, Germany).

b) EH TR-FIA. This assay was performed in a similar format to that of ETH. Biotinylated EH was prepared by incubating 15 nmol EH, dissolved in 100 µl 0.1 M sodium borate buffer pH 8.2 with 150 nmol EZ-Link® NHS-LC biotin (Thermo Scientific, Rockford, IL, USA) overnight on ice with constant stirring. The biotinylated product was purified on a SPE column (Strata-X), eluting biotinylated peptide with 60% acetonitrile, and drying by vacuum centrifugation followed by reconstitution in assay buffer to give a nominal concentration of 10 nmol/ml. Standard EH concentrations were 1000–2 fmol/50 µl, biotinylated EH 125 fmol/50 µl, affinity purified anti EH IgG 25 ng/50 µl. Detection limit: 2–5 fmol EH/well.

c) Ecdysteroid radioimmunoassay (RIA).

Ecdysteroid RIA was performed using the following conditions: 100 µl 1/2000 anti-ecdysteroid serum HB-2E, 100 µl H^−3^ Ponasterone A (24, 25, 26, 27-3H (N), 3.5 TBq mmol^−1^ (American Radiochemical Company, St. Louis, MO, USA), 28,000 dpm/tube. 25-deoxyecdysone was used as a standard (Range 2500–19.5 pg/tube), standards and unknowns were assayed in triplicate. All reagents were diluted in 0.1 M sodium borate buffer, pH 8.2. Bound was separated from free using 50 µl/tube immobilized donkey anti-rabbit microcellulose suspension (Sac-Cel® Immunodiagnostic Systems, Boldon, Tyne and Wear, UK). Pellets were resuspended in 600 µl Hi-Safe 3 (PerkinElmer) prior to scintillation counting. Using this RIA, both ecdysone and 25-deoxyecdysone (the two ecdysteroids synthesized by *C. maenas* Y-organs) were equally recognized. Detection limit: 20 pg/tube.

Hemolymph samples from molt-staged crabs for assay were purified by extraction in 75% methanol, centrifugation, drying in a vacuum centrifuge and reconstitution in 100 µl borate buffer.

### Receptor cloning cell culture and receptor assays

Transcriptome data from *C. maenas* [38] were queried for identity to ETHR’s functionally deorphaned in insects. A single candidate was identified (TR677726).

The putative ETH receptor was synthesized and subsequently cloned into pcDNA3.1 + via EcoR1-Not 1 (GenScript, Piscataway, NJ, USA). The sequence included a modified Kozak sequence (GAATTCGCCACC). Plasmid vectors were transformed into chemically competent OneShotTOP10® *E. coli* (Invitrogen, Paisley, UK) and grown on LB plates containing 100 µg ml^−1^ ampicillin at 37 °C overnight. Individual colonies were verified by PCR, and positive clones expanded by overnight incubation in LB broth (37 °C, overnight). Plasmids were isolated using a MidiPrep kit (QIAGEN GmbH, Hilden, Germany) according to the manufacturer’s instructions, resequenced (MWG Eurofins, Ebersberg, Germany) and analyzed using Geneious V 9.1.8 [65].

Chinese hamster ovary (CHO-K1) cells containing stably expressed apoaequorin (PerkinElmer) and either Gα16 or G*q* Subunit (control cells) were cultured in Dulbecco’s Modified Eagle Medium (DMEM) F-12 Nutrient Mixture Glutamax (Gibco®) supplemented with 10% fetal bovine serum (Gibco®). Cells were maintained in vented T75 flasks at 37 °C in 5% CO_2_. Cells grown in a monolayer to approx. 60% confluency were transfected with pcDNA 3.1 constructs using FugeneHD® (Promega). Transfection medium was prepared by combining 800 μl Opti-MEM® (Gibco) with 10 µg vector and 30 μl Fugene HD in a 2 ml polystyrene tube and incubated for 10 min at room temperature. The transfection reagent was added to 5 ml of fresh culture media and the cells incubated overnight at 37 °C in 5% CO_2_. After 24 h, an additional 10 ml of culture media were added to the flasks and the cells returned to 37 °C at 5% CO_2_ for a further overnight incubation.

Cells were detached from the culture flask by incubating for 10 min in 5 ml 0.2% EDTA in PBS, washed in 10 ml clear DMEM/F-12 containing L-glutamine, 15 mM HEPES and centrifuged (5 min, 260 g), resuspended at a concentration of 5 × 10^6^ cells/ml in 0.2-µm filtered BSA medium (DMEM/F-12 with L-glutamine, 15 mM HEPES, 0.1% BSA). Coelenterazine *h* (Invitrogen, Paisley, UK) was added to give a concentration of 5 µM, and the cells incubated in the dark at RT with gentle rocking. Before the assay, cells were further diluted (tenfold) and incubated for 60 min before use. Synthetic peptides used in the assay are listed (Additional file 3: Table 1). Dried aliquots of peptides were reconstituted in BSA medium, and 50 µl added in quadruplicate to a white 96-well plate (Optiplate®, PerkinElmer). Cell suspensions were gently stirred and injected (50 µl/well) using a Mithras LB 940 microplate reader (Berthold Technologies, Bad-Wildbad, Germany), and light emission (Ca^2+^ response recorded for 30 s). Cells were then lysed by injection of 50 µl/well 0.3% Triton-X in BSA medium, and light emission recorded for a further 10 s to measure total Ca^2+^ response. BSA medium was used for blank measurements (6 replicates per plate), and transfection with empty vectors used for negative controls. Data was analyzed using Mikrowin v5.18 (Mikrotek Laborsysteme GmbH) and SigmaPlot v15 (Systat Software Inc, Grafiti, Slough, UK).

To determine whether ETH is additionally signaled via cAMP, the following assay was used. HEK293 cells in DMEM (Gibco) supplemented with 10% FBS (Sigma) were seeded at a density of 2 × 10^4^ cells/well in a 96 well tissue culture plate and incubated for 18–20 h at 37 °C and 5% CO_2_ prior to transfection. Cells were cotransfected with CamETHR pcDNA3.1 plasmid and a Cre-luciferase reporter construct (pGL4 CRE-CMVmin-luc2, Addgene) using FuGene® 4 K transfection reagent (Promega) according to the manufacturer’s recommendations. Briefly, 3 ml of Opti-MEM was combined with 4 µg of receptor plasmid and 27 µl FuGene 4 K in a low bind polystyrene tube and incubated for 20 min at RT. DMEM culture medium was replaced with 30 µl of transfection cocktail in each well in a 96 well microplate and incubated at 37 °C in 5% CO_2_ for 2 h. A further 60 µl of culture medium was added before incubation overnight. The incubation medium was replaced with serum-free DMEM 17–18 h prior to assay.

Synthetic ETH was redissolved in DMEM without phenol red (Gibco) and 100 µl aliquots dispensed into a 96 well microplate in sextuplicate. DMEM was used for blank measurements, and the cells incubated for 4 h at 37 °C. Luciferase was measured using a SteadyLite Plus High Sensitivity Luminescence Reporter Gene Assay system (PerkinElmer), replacing the ligand media with 100 µl DMEM and an equal volume of SteadyLite reagent. The plate was mixed and incubated in darkness for 15 min. The cell lysates were transferred to a white 96-well plate (Optiplate, PerkinElmer) and luminescence measured for 1 s/well at 20 °C on a Mithras LB 940 plate reader and analyzed using MikroWin.

### HPLC and MS

EH containing fractions were obtained by HPLC of dissected medulla eyestalk tissues, extracted in ice-cold 2 M acetic acid via sonication, centrifuged and dried by vacuum centrifugation. Dried extracts were reconstituted in 2 M acetic acid and separated by HPLC. Conditions were Jupiter 250 × 4.8 mm 300 Å column (Phenomenex, Macclesfield, UK), solvent A: 0.11% trifluoroacetic acid (TFA), B: 0.1% TFA, 60% acetonitrile, 20–80% B over 40 min, 1 ml/min. For ETH separation. SPE purified samples of C4 and E90 molt stage hemolymph were first purified by SPE as detailed earlier. Dried peptide fractions were reconstituted in 2 M acetic acid and chromatographed as detailed, but with a gradient of 30–80% B over 40 min. Samples of HPLC purified immunopositive material were analyzed for mass by electrospray-time of flight mass spectrometry (ES-TOF MS) by Alta Bioscience Ltd, Redditch, UK.

### End point and digital droplet PCR

Total RNA was extracted from frozen tissues using TRIzol (Invitrogen, Carlsbad, USA) according to the manufacturer’s instructions, followed by removal of gDNA (TURBO DNA-free, Invitrogen), and mRNA selection using Dynabeads Oligo (dT)_25_ (Dynal, Oslo, Norway) and stored in 10 mM Tris–HCl at − 80 °C. cDNA synthesis was carried out using a Tetro cDNA synthesis kit (Bioline, Meridian Bioscience) using a mix of random hexamers and oligo (dT)_18_ primers, and the following conditions: 25 °C 10 min, 45 °C 30 min, 85 °C 5 min. cDNA samples from stage D4 tissues were subjected to end point PCR to determine ETH and ETHR transcript distribution. PCR was performed in 25 µl volumes using Dream Taq® DNA polymerase (Thermo Fisher Scientific, Vilnius, Lithuania). 98 °C 5 min, followed by 35 cycles 95 °C 30 s, 58 °C 30 s, 72 °C 45 s, final extension 72 °C 5 min (ETH) or 40 cycles (ETHR) primers used are detailed (Additional file 3). Products were electrophoresed on 2% agarose gels.

Digital droplet (dd)PCR was performed on molt staged nervous system cDNA produced as detailed above. PCR reactions were performed using ETH primers and TaqMGB probe sets for ETH and Ubiquitin-conjugating enzyme E2 L3 *(UBE2L3)* (Additional file 3: Table 1) and as detailed [38]. Duplex reactions were assembled in 20 µl volumes using ddPCR® Supermix for Probes (Bio-Rad, Hercules, California USA) with 1 µM each primer and 250 nM each probe (ETH, VIC and UBE2L3) and 1 µl of cDNA (stock diluted 10 ×). Droplets were then generated in a Bio-Rad QX200® Droplet Generator according to the manufacturer’s instructions before sealing the plate (Bio-Rad PX1 plate sealer) and thermocycling. Conditions were: 95 °C, 10 min, 94 °C 30 s, 60 °C 60 s for 40 cycles, final extension 95 °C, 10 min, 4 °C until the droplets were read. Droplet fluorescence, quantification, and analysis was performed on a Bio-Rad QX200® droplet reader with QuantaSoft® proprietary ddPCR analysis software. ETH quantification was normalized against *UBEL2L3* as the reference gene. *UBEL2L3* has previously been shown to be constitutively expressed across all molt stages with RNASeq analysis [38].

## Supplementary Information

Additional file 1: Figure S1. Immunohistochemistry; preabsorption controls for ETH and EH.

Additional file 2: Figure S2. HPLC TR-FIA of CNS extract; ETH confirmation, HPLC TR-FIA and MS of eyestalk extract; EH confirmation.

Additional file 3: Table 1. Primer and peptide sequences used.

Additional file 4: Figure S3. 2D barrel model of Cam ETHR. 

Additional file 5: Figure S4. Phylogram of putative and functionally deorphanised ETHR homologs in selected insects and crustaceans. 

Additional file 6: Figure S5. Sequence alignments and phylogram of eclosion hormone-like peptides in selected decapod crustaceans. 

Additional file 7: Figure S6. Raw images of agarose gels. 

## Data Availability

The datasets used and analyzed during the current study and materials generated (e.g. antisera, peptides, plasmids) are available from the corresponding author on reasonable request. ETH, ETHR and EH sequences have been deposited in GenBank under the following accession numbers: Cam EH-1, PX841030; Cam ETH, PX841031; Cam ETHR, PX841032.

## Abbreviations

AN: Antennary nerve
Arg 8VP: Arginine vasopressin
AST-C: Allatostatin-C
BSA: Bovine serum albumen
CCAP: Crustacean cardioactive peptide
CCAPR: Crustacean cardioactive peptide receptor
cAMP: Cyclic 3′ 5′ adenosine monophosphate
cGMP: Cyclic 3′ 5′ guanosine monophosphate
CRE-Luc: CAMP response element-luciferase
CAP2B: Cardioactive peptide 2B
CG: Cerebral ganglion
CHO: Chinese hamster ovary
CNS: Central nervous system
COC: Circum-oesphageal connectives
COG: Commissural ganglion
CPK: Carcipyrokinin
CRZ: Corazonin
ddPCR: Droplet digital Polymerase Chain Reaction
DH: Diuretic hormone
DMEM: Dulbecco’s modified eagle medium
dsRNAi: Double-stranded RNA interference
EH: Eclosion hormone
ES: Eyestalk
ETH: Ecdysis triggering hormone
ES-TOF MS: Electrospray-time of flight mass spectrometry
ETHR: Ecdysis triggering hormone receptor
FBS: Fetal bovine serum
GLM: Generalized Linear Model
GPCR: G protein-coupled receptor
HCR-FISH: Hybridization chain reaction-fluorescent in situ hybridization
HEK: Human embryonic kidney
HPLC: High-performance liquid chromatography
IHC: Immunohistochemistry
ISH: In situ hybridization
mRNA: Messenger ribonucleic acid
MIPs: Myoinhibitory peptides
ON: Optic nerve
Oxy: Oxytocin
PAP/DAB: Peroxidase anti-peroxidase/diaminobenzidine
PETH: Pre-ecdysis triggering hormone
PDH: Pigment dispersing hormone
PBS: Phosphate buffered saline
PFA: Paraformaldehyde
PO: Pericardial organ
RNA: Ribonucleic acid
RPCR: Red pigment concentrating hormone
RT: Room temperature
RT-PCR: Reverse transcription polymerase chain reaction
RT: Room temperature
SA: Sternal artery
SCPB: Small cardioactive peptide 2B
SOG: Subesophageal ganglion
SPE: Solid phase extraction
TC: Tritocerebrum
TFA: Trifluoroacetic acid
TG: Thoracic ganglia
TR-FIA: Time resolved-fluorescence immunoassay
UBE2L3: Ubiquitin-conjugating enzyme E2 L3
VG: Ventral ganglion
Vm: Ventromedial
YO: Y-organ
