# Supplementary material for: Identification of neuropeptide networks involved in the ecdysis program of a crustacean model: Carcinus maenas reveal similarities and differences to insects that reflect evolutionary divergence in structure and function
Source: BMC Biol. 2026 Apr 22;24:134. doi: 10.1186/s12915-026-02603-w (PMC13234976; doi:10.1186/s12915-026-02603-w)
Supplement: Supplementary file 1 — Additional file 1: Figure S1. Immunohistochemistry; preabsorption controls for ETH and EH. [file 12915_2026_2603_MOESM1_ESM.docx]

**
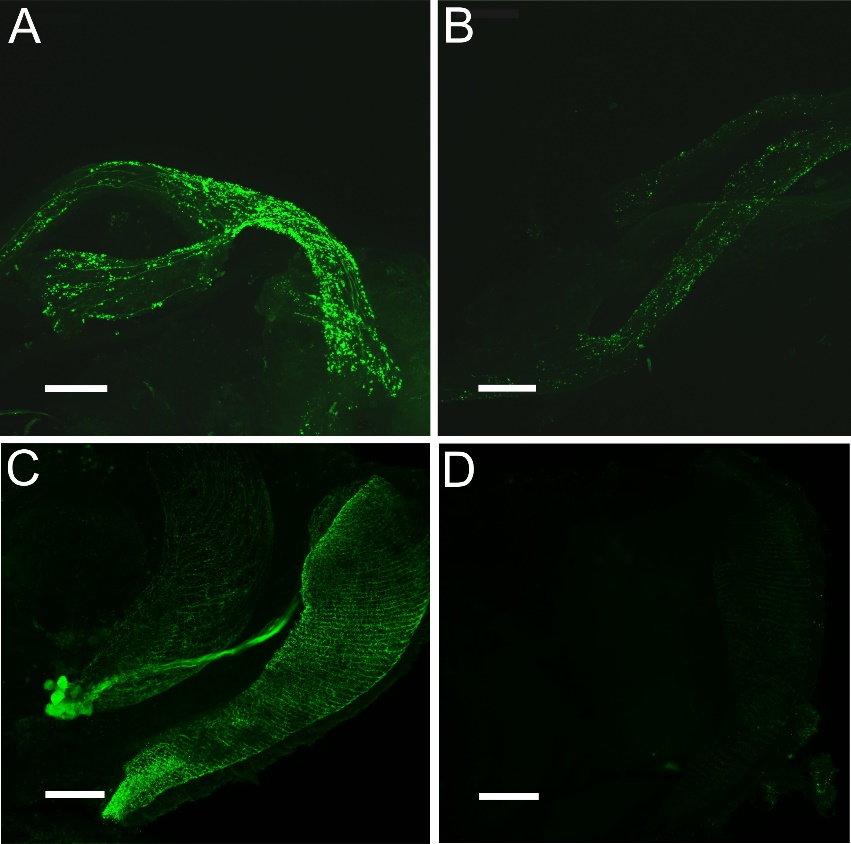
Additional file 1: Figure S1.**

Preabsorption controls for ETH (A, B) and EH (C, D)

1µL affinity purified anti-ETH IgG (10pmol) in 100µl PTX was incubated overnight (ON) 4^o^C without/with100pmol ETH and subsequently diluted 20-fold. Whole mount preparations of PO were incubated for 48h at 4^o^C, washed extensively in PTX, incubated ON in 1: 750 Alexa 488 goat anti-rabbit IgG washed and mounted. Z-stacked Confocal images were taken at identical attenuations, and then both adjusted equally for maximum but identical saturation (CorelDraw). A) Control, B) Preabsorption sample.

The procedure given above was followed for affinity purified anti-EH IgG, and synthetic EH. The antiserum was raised against linear EH. C) Control, D) Preabsorption sample. Scale bars 200µm.
