# Supplementary material for: Identification of neuropeptide networks involved in the ecdysis program of a crustacean model: Carcinus maenas reveal similarities and differences to insects that reflect evolutionary divergence in structure and function
Source: BMC Biol. 2026 Apr 22;24:134. doi: 10.1186/s12915-026-02603-w (PMC13234976; doi:10.1186/s12915-026-02603-w)
Supplement: Supplementary file 2 — Additional file 2: Figure S2. HPLC TR-FIA of CNS extract; ETH confirmation, HPLC TR-FIA and MS of eyestalk extract; EH confirmation. [file 12915_2026_2603_MOESM2_ESM.docx]

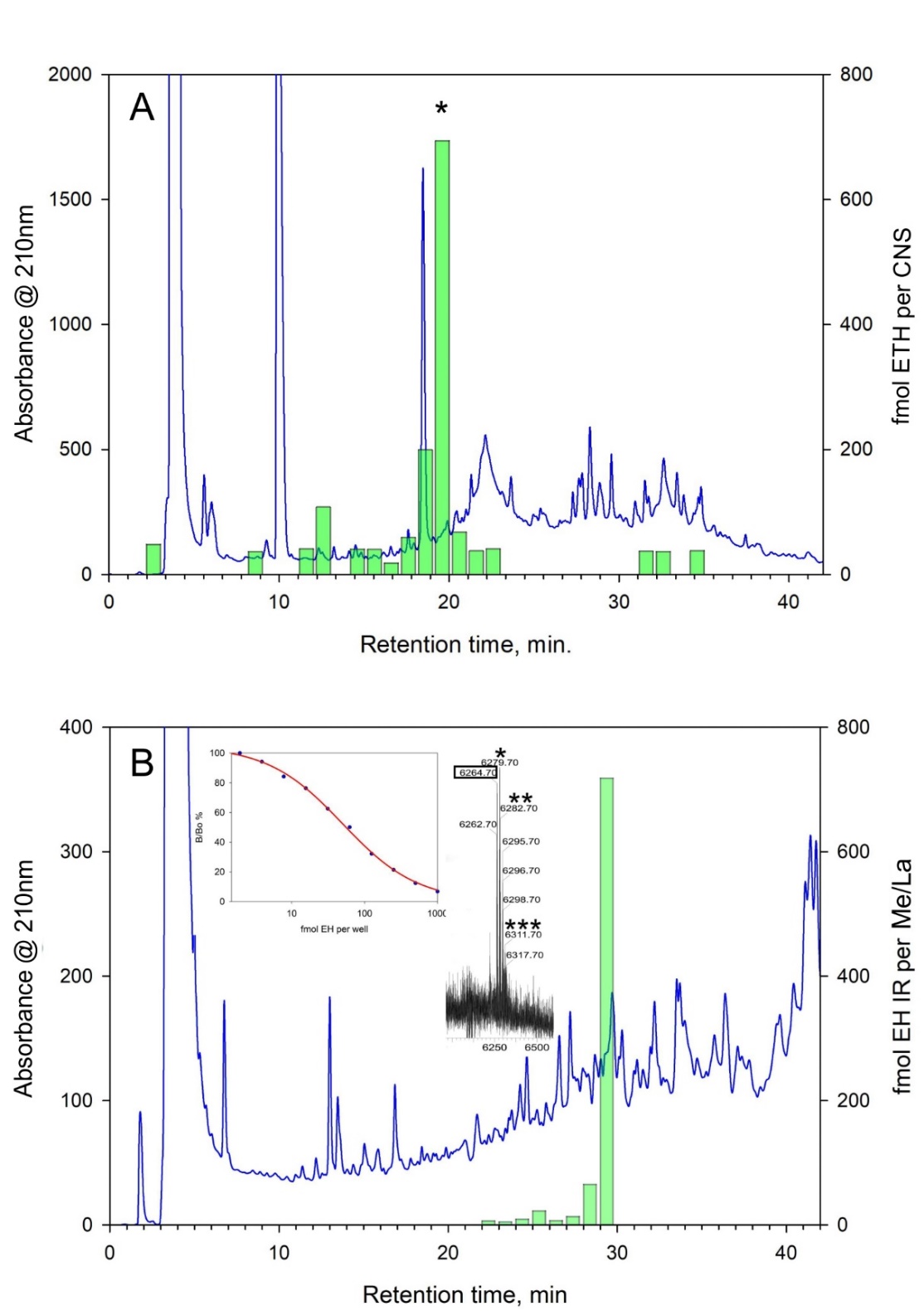
**Additional file 2: Figure S2.**

A) HPLC TR-FIA of a Strata-X purified extract of 3 Stage C4 CNS. Chromatographic conditions described in the text. Asterisk denotes retention time of an injection of 1pmol synthetic ETH.

B) HPLC TR-FIA of a 2M acetic acid extract of 4 medullae and laminae (Me/La) from Stage C4 crabs. Chromatographic conditions as in A). Inserts show a typical dose response curve, and MS (ES-TOF) of a sample of immunopositive material showing masses corresponding to native (boxed) and 1-3 Met sulfoxide adducts (asterisks).
