# Supplementary material for: Identification of neuropeptide networks involved in the ecdysis program of a crustacean model: Carcinus maenas reveal similarities and differences to insects that reflect evolutionary divergence in structure and function
Source: BMC Biol. 2026 Apr 22;24:134. doi: 10.1186/s12915-026-02603-w (PMC13234976; doi:10.1186/s12915-026-02603-w)
Supplement: Supplementary file 3 — Additional file 3: Table 1. Primer and peptide sequences used. [file 12915_2026_2603_MOESM3_ESM.docx]

**Additional file 3: Table 1.**

Primers used in PCR (A), *in- situ hybridization* (B) and ddPCR (C).

| **Primer Name** |  | **Sequence 5’-3’** |
| --- | --- | --- |
| CamETH F | A | GGCACAGGTGTGAGGACTAGT |
| CamETH R | A, B | CAATAGTGCTCGACCCTCACT |
| CamETH F T7 | B | TAATACGACTCACTATAGGGGGCACAGGTGTGAGGACTAGT |
| CamETHr 331 F | A | GGCACAGGTGTGAGGACTAGT |
| CamETHr 765 R | A | CAATAGTGCTCGACCCTCACT |
| EH 587 R | B | AGAGAAAAAGAAACACCACTCG |
| EH 107 F T7 | B | TAATACGACTCACTATAGGGAGCACTCTCACATCAACACACT |
| Cam ETH F TaqMan | C | GGCGGGCCTGGACAGT |
| Cam ETH R TaqMan | C | GGGATACGTAGCAGCTCTCCAA |
| Cam ETH-VIC | C | CGGAGACGGTTGTATTG |
| Cam UBQ F TaqMan | C | TCACCTGGCAGGGACTCATT |
| Cam UBQ R TaqMan | C | CCTGAACGCTCCCTTGTTGT |
| Cam UBQ-FAM | C | ACCCGAGAACCCACC |

Sequences of peptides used in ETHR assays. C- terminal PRX-amides or PLG-amides

are highlighted.

| CAP2_B_ | | pQGLYFAPRV-NH_2_ |
| --- | --- | --- |
| SCP_B_ | | MNYLAFPRM-NH_2_ |
| Arg (8) vasopressin | | CYFQNCPRG-NH_2_ |
| Oxytocin |  | CYIQNCPLG-NH_2_ |
| Carcipyrokinin 1 |  | LYFAPRL-NH_2_ |
| Carcipyrokinin 2 |  | DTGFAFSPRL-NH_2_ |
| Carcipyrokinin 3 |  | TSFAFSPRL-NH_2_ |
| *Carcinus* ETH |  | DAGHFFAETPKHLPRI-NH_2_ |
| *Carcinus* Y_5_ETH |  | DAGHYFAETPKHLPRI-NH_2_ |
| *Carcinus* A_1_ETH |  | ADAGHFFAETPKHLPRI-NH_2_ |
| *Locusta* ETH |  | SDFFLKTAKSVPRI-NH_2_ |
| *Periplaneta* ETH |  | SEYDNFFLKASKSVPRI-NH_2_ |
| *Apis* ETH |  | EPVAFFLKTAKSVPRV-NH_2_ |
| *Bombyx* PETH |  | SFIKPNNVPRV-NH_2_ |
