# Supplementary material for: Identification of neuropeptide networks involved in the ecdysis program of a crustacean model: Carcinus maenas reveal similarities and differences to insects that reflect evolutionary divergence in structure and function
Source: BMC Biol. 2026 Apr 22;24:134. doi: 10.1186/s12915-026-02603-w (PMC13234976; doi:10.1186/s12915-026-02603-w)
Supplement: Supplementary file 4 — Additional file 4: Figure S3. 2D barrel model of Cam ETHR. [file 12915_2026_2603_MOESM4_ESM.docx]

**Additional file 4: Figure S3.**


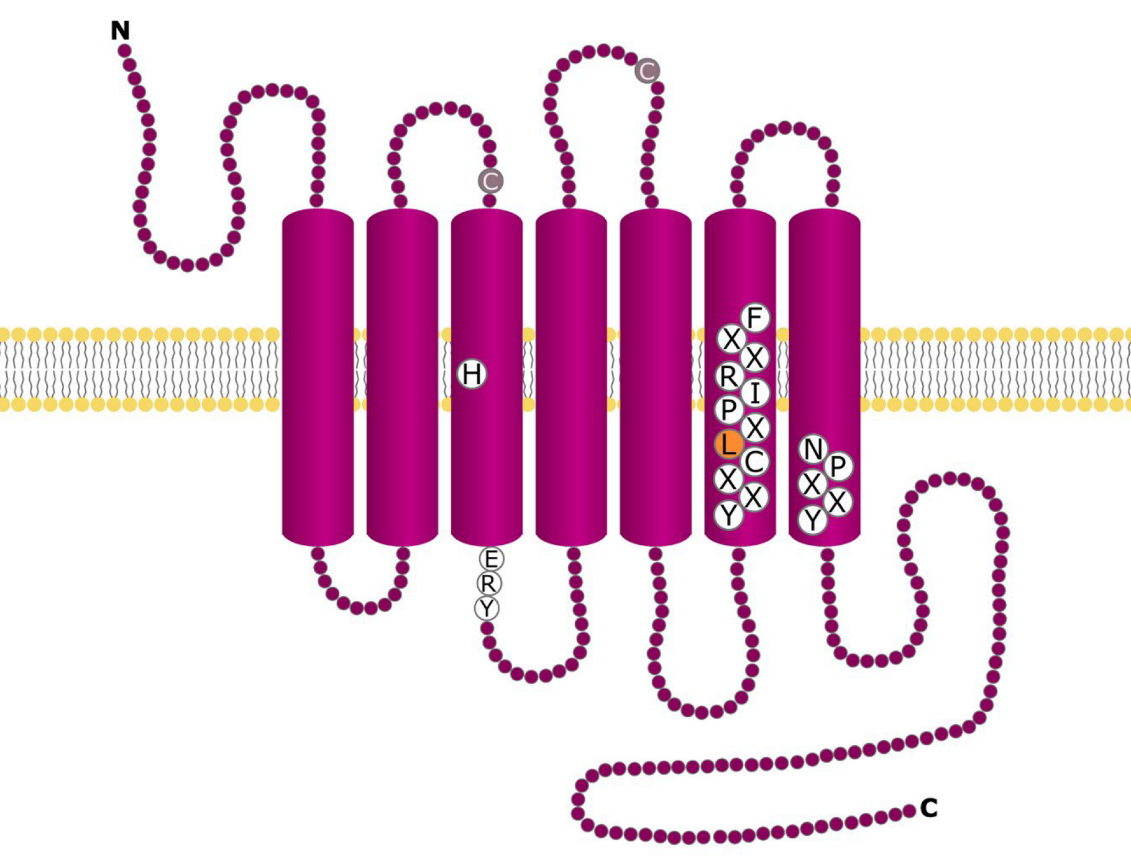
2D barrel model of Cam ETHR

The ERY motif on the intracellular side of TM3 is characteristic of PRXamide receptors and highly specific for ETH receptors. Another conserved region in TM6 of PRXamide receptors, (FXXCWXPFHXXR) has a specific modification from the tryptophan (W) residue to leucine (L) (orange) which is unique to the ETH receptors. The corresponding sequence YFLCLLPIRVFF in the *Carcinus* ETHR is shown with differences marked (X). The NPXXY motif near the intracellular side of TM7 is common to nearly all GPCRs. The histidine (H) residue in TM 3 is found in all ETH receptors The highly conserved cysteine (C) residues in extracellular loops 2 and 3, necessary for the formation of a disulfide bridge are shown (grey labels).
