# Supplementary material for: Identification of neuropeptide networks involved in the ecdysis program of a crustacean model: Carcinus maenas reveal similarities and differences to insects that reflect evolutionary divergence in structure and function
Source: BMC Biol. 2026 Apr 22;24:134. doi: 10.1186/s12915-026-02603-w (PMC13234976; doi:10.1186/s12915-026-02603-w)
Supplement: Supplementary file 5 — Additional file 5: Figure S4. Phylogram of putative and functionally deorphanised ETHR homologs in selected insects and crustaceans. [file 12915_2026_2603_MOESM5_ESM.docx]

**Additional file 5: Figure S4.**


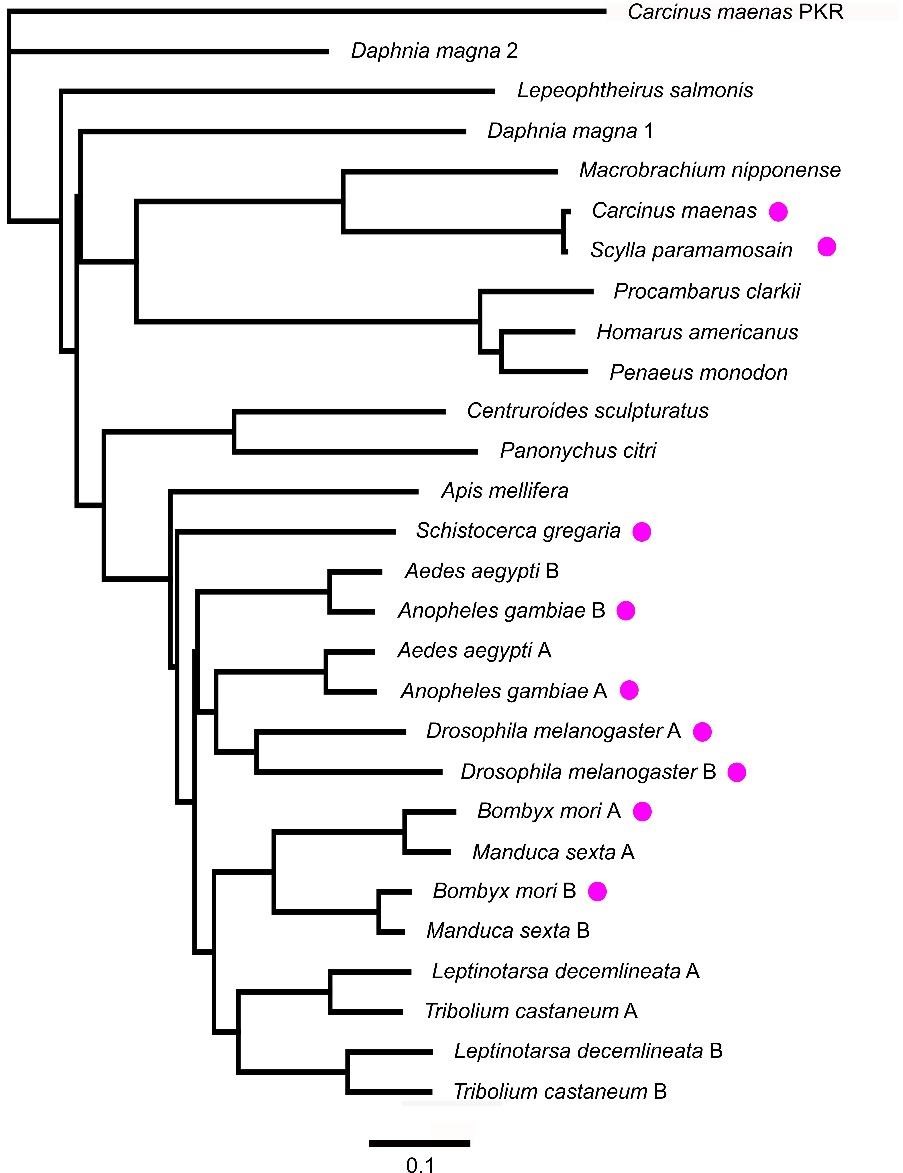


Phylogram of putative and functionally deorphanised (magenta circles) ETHR homologs in selected insects and crustaceans. Outgroup: *Carcinus maenas* pyrokinin receptor, (TR 181437, [46]).

*Daphnia magna* 1,2, [53]; *Lepeophtheirus salmonis*, (CDW44801.1); *Macrobrachium nipponense*, (KU532132.1); *Carcinus maenas* (TR677726, [46]); *Scylla paramamosain*, (WCZ69641.1, [55]); *Procambarus clarkii* (XP_045618224); *Homarus americanus* (QPB70556.1); *Penaeus monodon* (XP_037795426.1); *Centruroides sculpturatus* (XP_023240730.1); *Panonychus citri* (AZL90164.1); *Apis mellifera* ( (XP_006570143.1); *Schistocerca gregaria* (ARK38543.1, [51]); *Aedes aegypti* A, B (ABI93273.1, ABI93274.1); *Anopheles gambiae* A, B (QXN57742.1, QXN57743.1); *Drosophila melanogaster* A,B (AAF55782.1, NP_996255.1*); Bombyx mori* A, B (NP_001165737.1, NP_001165737.1); *Manduca se*xta A,B (AAX19163.1, AAX19164.1); *Leptinotarsa decemlineata* A,B (QBH70335.1, QBH70336.1), *Tribolium castaneum* A, B (ABN79653.1, ABN79654.1).

Sequences were trimmed to include only the predicted 7-TM and loop domains. Phylograms were assembled using Geneious V.8 tree builder, using a Jukes-Cantor model with the neighbor joining default setting.
