# Supplementary material for: Identification of neuropeptide networks involved in the ecdysis program of a crustacean model: Carcinus maenas reveal similarities and differences to insects that reflect evolutionary divergence in structure and function
Source: BMC Biol. 2026 Apr 22;24:134. doi: 10.1186/s12915-026-02603-w (PMC13234976; doi:10.1186/s12915-026-02603-w)
Supplement: Supplementary file 6 — Additional file 6: Figure S5. Sequence alignments and phylogram of eclosion hormone-like peptides in selected decapod crustaceans. [file 12915_2026_2603_MOESM6_ESM.docx]

**Additional file 6: Figure S5.**


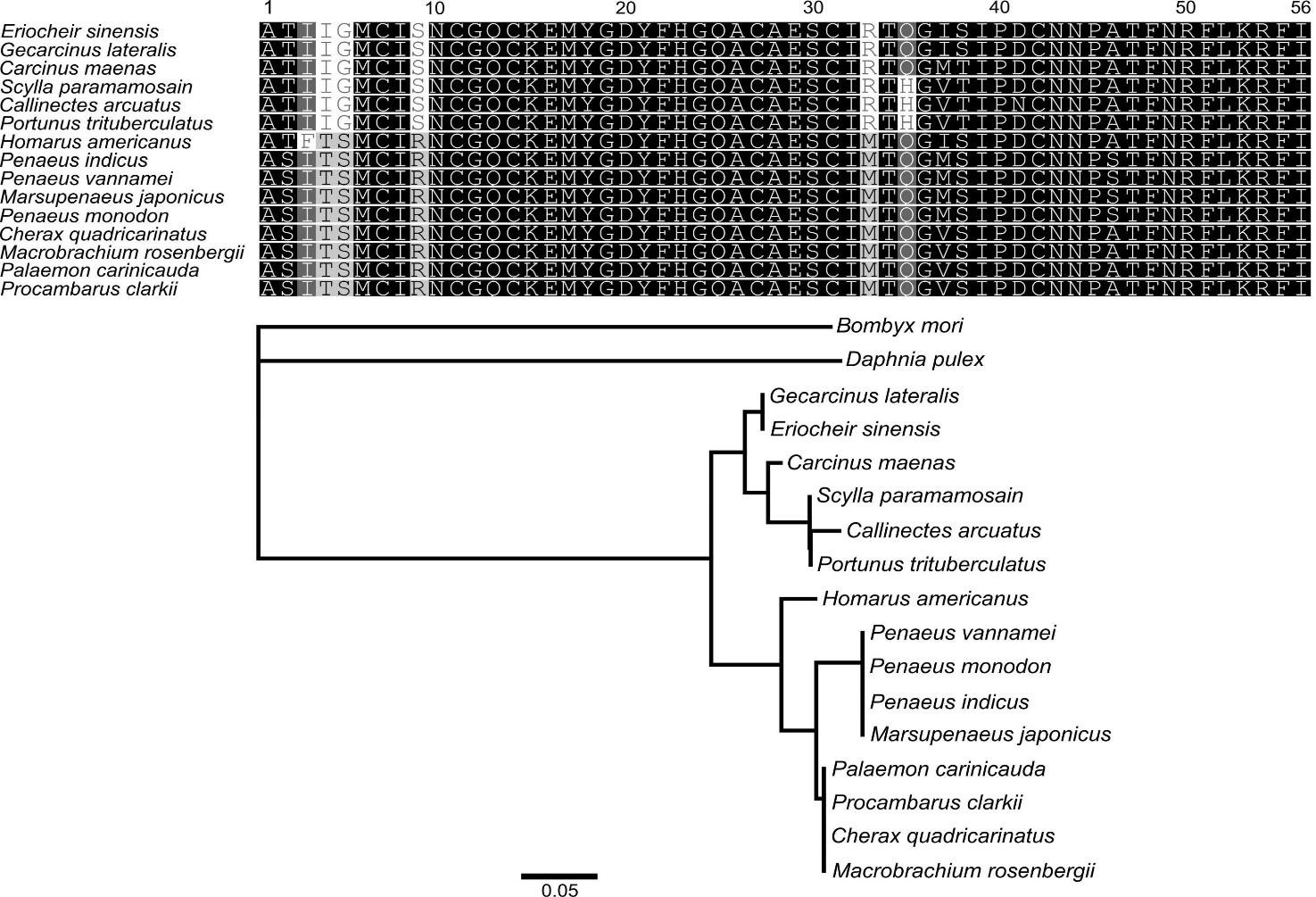
.

Sequence alignments (mature peptide) and phylogram (including prepro-peptide) of eclosion hormone- like neuropeptides in selected decapod crustaceans. Phylograms were assembled using Geneious V.8 tree builder using a Jukes-Cantor model with the neighbour joining default setting. Accession numbers are as follows: *Eriocheir sinensis*, XP_027227955.1; *Gecarcinus lateralis*, WLV89615.1; *Carcinus maenas* (PX841030); *Scylla paramamosain*, ALQ28581.1; *Callinectes arcuatus*, QP025062; *Portunus trituberculatus*, XP_045136966.1; *Homarus americanus*, KAG7170701; *Penaeus indicus*, XP_063593827.1; *Penaeus vannamei*, XP_027227955.1; *Marsupenaeus japonicus*, XP_042875065; *Penaeus monodon*,

XP_037791859.1; *Cherax quadricarinatus*, XP_053627890.1; *Macrobrachium rosenbergii*, XP_066968456.1; *Palaemon carinicauda*, XP_068244715.1. Outgroups: *Bombyx mor*i, NP_00137307.1; *Daphnia pulex*, EFX83453.1.
