# Supplementary material for: Identification of neuropeptide networks involved in the ecdysis program of a crustacean model: Carcinus maenas reveal similarities and differences to insects that reflect evolutionary divergence in structure and function
Source: BMC Biol. 2026 Apr 22;24:134. doi: 10.1186/s12915-026-02603-w (PMC13234976; doi:10.1186/s12915-026-02603-w)
Supplement: Supplementary file 7 — Additional file 7: Figure S6. Raw images of agarose gels. [file 12915_2026_2603_MOESM7_ESM.docx]

**Additional file 7. Figure S6.**

**
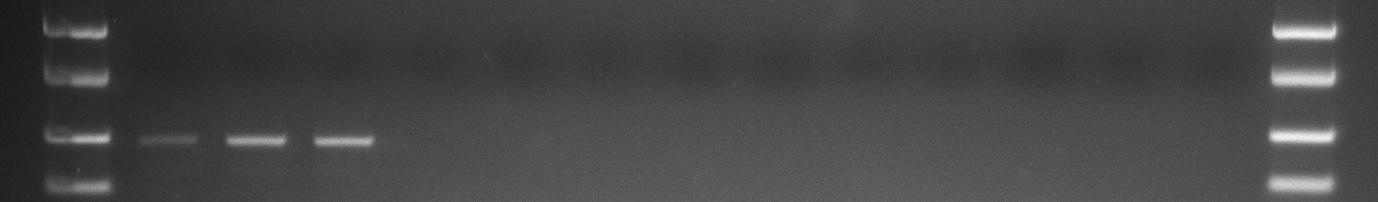
**

**
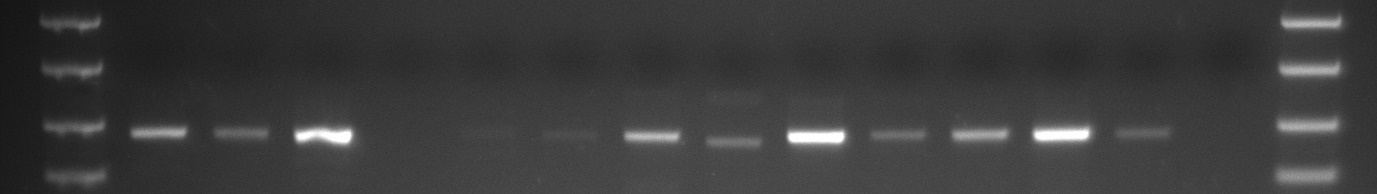
**

Raw uncropped images of agarose gels for ETH PCR tissue panel (upper) and ETHR tissue panel (lower). See Fig. 2 for tissue identifiers.
